# Developing age-friendly spaces through a gerontechnological lens: a systemic framework based on FDM-DANP analysis

**DOI:** 10.3389/fmed.2025.1681486

**Published:** 2025-10-13

**Authors:** Wei-Quan Zheng, Sze-Man Cheung, Xi Wang

**Affiliations:** ^1^Faculty of Humanities and Social Sciences, Macao Polytechnic University, Macau, Macao SAR, China; ^2^Graduate Institute of Building and Planning, National Taiwan University, Taipei, Taiwan

**Keywords:** age-friendly, Gerontechnology, multi-criteria decision-making (MCDM), systematic integration, spatial design

## Abstract

With the acceleration of population aging and the spread of smart technologies, integrating Gerontechnology with spatial design has become an urgent challenge. Although previous studies have examined technology acceptance and environmental adaptation, they have lacked a systematic framework to capture multiple dimensions and their interdependencies with empirical evidence. This study proposes a two-stage integrated framework that combines the Fuzzy Delphi Method (FDM) and the Decision-Making Trial and Evaluation Laboratory-based Analytic Network Process (DANP) to identify and quantify the key factors and causal structures of integrating Gerontechnology into spatial design. This study utilized FDM to screen out 15 key indicators. The DANP results show that within “Age-Friendly Design,” *C*_10_: Fault-tolerant spatial configuration received the highest weight (global weight 26.42%), followed by *C*_9_: Comfortable ambient temperature under “Living Space” (7.18%). These findings highlight the central role of fault-tolerant spatial configuration and environmental comfort in the integrated framework. In DANP, the consensus index of experts for all dimensions exceeded 95%, confirming the robustness of the findings. In addition, the DEMATEL results reveal that Gerontechnology Application has a primary driving effect on other dimensions and elements in the causal network, showing its key role in system integration. These findings provide policymakers and practitioners with clear references for prioritization and spatial planning, and also offer actionable decision support for smart spatial strategies and cross-sector collaboration in the context of healthy aging.

## 1 Introduction

Technological and medical innovations have emerged as key contributors to life extension, enhancing overall social welfare and facilitating the transition toward healthy aging. This trend has introduced new challenges in the allocation of healthcare and caregiving resources, while simultaneously generating institutional and technological demands in fundamental domains such as housing, mobility, and social participation. Previous studies have suggested that smart cities represent a promising approach for addressing the societal implications of population aging (1). Gerontechnology, in particular, is widely recognized as a foundational component in enabling healthy aging within the framework of smart cities (2, 3).

Gerontechnology refers to the interdisciplinary integration of gerontology and technological sciences, aimed at promoting the health, independence, and overall life satisfaction of older adults (4–8). Numerous studies have demonstrated that the effective integration of Gerontechnology into the daily lives of older adults can improve their functional independence, mitigate physical decline, and enhance social engagement (9, 10). However, despite the widely acknowledged potential of technology and the ongoing development of Gerontechnology devices and services, older adults continue to experience significant barriers to technology adoption compared to younger or more digitally literate populations (11, 12). Meanwhile, older adults continue to report low levels of technology acceptance and user satisfaction in everyday living contexts (13–15). Peek et al. (16) emphasized that the compatibility and adaptability between technology and the physical environment critically shape older adults’ willingness to adopt Gerontechnology. For instance, a mismatch between road safety conditions and safety technologies may reduce their acceptance of transportation-related Gerontechnology. Moreover, an increasing body of research has highlighted older adults’ reservations and skepticism regarding the misalignment between technology and environmental contexts (119, 120). This suggests that in the design of age-friendly environments, there remain challenges related to the lack of synchronization between Gerontechnology systems and spatial infrastructures, leading to a disconnection between Gerontechnology Applications and everyday living contexts (17, 18). Therefore, in response to the growing demand for age-friendly environments, identifying the key factors that influence the integration of Gerontechnology into physical spaces is of critical importance.

In recent years, Multi-Criteria Decision-Making (MCDM) methods have been extensively applied in studies on age-friendly environments and facility design. For instance, Jiravanichkul et al. (19) employed the Analytic Hierarchy Process (AHP) to develop evaluation criteria for Thailand’s Well-Being Environment and Age-Friendly Communities, emphasizing that local governments should account for the unique environmental needs of older adults in planning. Zarghami et al. (20) applied AHP to assess factors influencing quality of life in Iranian older adults housing, revealing that physical and perceptual aspects of design carried the greatest weight in decision-making. Similarly, Weck et al. (21) utilized Multiple Criteria Decision Aid (MCDA) to examine how to balance sustainability and age-friendly requirements in smart living environments. Collectively, these studies demonstrate that MCDM can provide a structured indicator system and a basis for decision support in age-friendly design. Nevertheless, existing research remains largely focused on single dimensions and lacks an analytical framework to systematically integrate Gerontechnology with the design of age-friendly spaces. At the same time, these studies have tended to overlook spatial environments as active components in the design process, without adequately addressing the need for coordinated spatial planning and structural compatibility. Sometimes, home modifications even led to unintended negative consequences for older adults, and in some cases, even exacerbated their living challenges (17, 18, 22–24). Therefore, establishing effective integration between spatial systems and Gerontechnology has become a pressing challenge for both academic researchers and industry practitioners.

Under the influence of the silver economy, both Gerontechnology-related industries and urban governance frameworks have begun to reassess the evolving lifestyles of older adults (25, 26). However, the current development of Gerontechnology remains largely product-driven, with design efforts heavily focused on ergonomics while overlooking the contextual realities of older adults’ living environments (2, 18). Simultaneously, prior research has tended to treat technology and Living Space as distinct domains, lacking comprehensive frameworks to examine their interrelations and overlooking the structural interdependence between spatial configurations and technological functionalities (14). In addition, prior studies have identified the lack of user-centered design as a primary barrier to the adoption of smart home systems, resulting in misalignment between users’ needs, capabilities, and the functionalities of available technologies (27, 28). Therefore, as technological systems grow increasingly complex, there is an urgent need to advance age-friendly research and ensure that its outcomes are effectively translated into the development of Gerontechnology (29).

Notably, the integration of Gerontechnology with spatial environments often entails substantial implementation and maintenance costs (30). Given the complexity of influencing factors, such financial burdens risk exacerbating the digital divide, potentially excluding segments of the older population from equitable access to smart living environments (29, 31). This underscores the need for age-friendly integration frameworks to incorporate considerations of resource allocation, offering decision-makers cost-effective models that translate into practical and affordable strategies for older adults. Therefore, the primary objective of this study is to investigate how Gerontechnology can be effectively integrated into specific living and spatial contexts to address the practical needs of older adults across key domains of age-friendly environments, including housing, health, and safety.

Accordingly, this study addresses the following research questions:

1. How can the key factors underlying the integration of Gerontechnology and spatial design be identified from an age-friendly perspective?
2. How can the causal relationships and relative influence among these factors be systematically modeled to develop a cost-effective and strategically meaningful integration framework?

To answer these questions, this study aims to identify the key factors involved in the integration of Gerontechnology and spatial environments, and to elucidate their causal structure and relative influence.

The methodology adopted in this study consists of the following steps: First, to identify key factors, the Fuzzy Delphi Method (FDM) is applied to refine potential indicators derived from the literature, resulting in an expert-validated framework. FDM is particularly effective in addressing high-cost issues by minimizing the allocation of resources to unnecessary or non-demand-driven components. This step lays the groundwork for developing integration strategies that are both feasible and affordable. Subsequently, the DEMATEL-based Analytic Network Process (DANP) is employed to analyze the interdependencies among the selected indicators. DEMATEL is well-suited to uncovering complex causal interactions among criteria, while ANP enables the prioritization of these factors, assisting decision-makers in resource allocation and strategic planning (32, 33). DEMATEL is well-suited to uncovering complex causal interactions among criteria, while ANP enables the prioritization of these factors, assisting decision-makers in resource allocation and strategic planning (32, 33). By grounding this framework in the convergence of everyday environments and technology, the study contributes to advancing the sustainable development and practical realization of smart cities in aging societies.

## 2 Literature reviews

### 2.1 Research on Gerontechnology

Pilotto et al. (8) and Parra-Rodríguez et al. (7) defined and classified Gerontechnology into three main domains: (i) Information and communication technologies (ICT), referring to tools that facilitate access to information and communication, such as digital platforms, telemedicine programs, and the application of artificial intelligence in healthcare; (ii) Assistive technologies aimed at preserving the independence and safety of older adults, including environmental monitoring systems, personal sensors, and smart home devices; and (iii) Human–machine interaction technologies that support the therapy and rehabilitation of older individuals with mobility or cognitive impairments, such as robotics, exergames, and virtual reality-based interventions, with demonstrated clinical benefits and potential to enhance social engagement. Prior research on Gerontechnology has predominantly relied on classical technology acceptance frameworks, particularly the Technology Acceptance Model (TAM) [e.g., (13, 34, 35)] and the Unified Theory of Acceptance and Use of Technology (UTAUT) [e.g., (15, 36–38)], to investigate various Gerontechnology use scenarios. In examining older adults’ engagement with technology, existing studies have primarily focused on individual-level psychological determinants (39, 40), including perceived usefulness (41), technology-related anxiety (42, 43), perceived ease of use (2), and behavioral intention (39).

The aforementioned studies have provided valuable insights into older adults’ intention to adopt Gerontechnology, offering useful implications for its future development and implementation. However, the adoption of technology among older adults is shaped not only by psychological determinants but also by contextual factors such as health conditions and specific technological needs (44). Melander-Wikman et al. (45) observed that older adults may be willing to compromise on privacy in exchange for enhanced mobility and safety, provided they retain autonomy over how such technologies such as alarm systems are used. This suggests that older adults’ acceptance behaviors may vary according to the function and nature of the technology. Therefore, the application of Gerontechnology should be guided by principles of selectivity and integration, thereby ensuring long term sustainability while maintaining cost effectiveness.

### 2.2 Spatial dimensions of aging

#### 2.2.1 Spatial environment

Space, as a socially constructed entity shaped by multiple environmental factors, plays an enduring role in shaping social relations, power dynamics, and structural inequalities (17, 46). As individuals age and their physiological and psychological needs evolve, spatial environments may give rise to new forms of age-related spatial inequality (47). For instance, the digital divide associated with the advancement of Gerontechnology may marginalize certain communities and populations by limiting their equitable access to smart environments (29). Against this backdrop, a growing body of research has highlighted the critical role of spatial environments in fostering inclusive and sustainable urban development, which in turn has significant implications for promoting healthy aging among older populations (17). On one hand, there is increasing awareness of the health-promoting benefits of Public Spaces for older adults, particularly the importance of high-quality public environments in mitigating loneliness and reducing the risk of social exclusion (48, 49). On the other hand, residential environments play a fundamental role in supporting the daily well-being and quality of life of aging individuals (50). Compared to other age groups, older adults tend to spend a greater proportion of their time within the home, making factors such as comfort, safety, and design quality in residential spaces directly influential on their overall well-being (50, 51).

Drawing on insights from health geography and environmental gerontology, earlier scholars began incorporating physical environments into aging research (16). For example, Melander-Wikman et al. (45) indicated that older adults’ adoption decisions may be shaped by mobility and safety related infrastructure, while Huang and Oteng (44) highlighted that community infrastructure and spatial support may play a critical role in enhancing Gerontechnology adoption. Nevertheless, prior studies have primarily focused on the influence of single environmental dimensions, without adequately addressing the potential synergies between Gerontechnology, the spatial contexts in which older adults live, and their associated spatial perceptions. Therefore, this study considers real life spatial contexts as a key dimension and, drawing on the concept of Gerontechnology, proposes a framework for space and technology integration grounded in real world contexts. This framework offers valuable insights into how Gerontechnology can be leveraged to promote active aging.

#### 2.2.2 Age-friendly design

Human-centric has been widely recognized as a foundational principle for enhancing the well-being, autonomy, and health-related rights of older adults (52). As aging societies evolve, human-centric approaches have been embedded in both geriatric care and technological development. Notable paradigms include relationship-centered care (53–56), person-centered care (54, 57, 58), and user-centric design (27, 59–61). User-centric design, in particular, emphasizes that the development of Gerontechnology must accommodate the diverse needs, abilities, and psychological responses of end users (27, 59). Failure to do so may result in technology rejection by older adults, often due to misalignments between the product’s functionalities and users’ perceptions, attitudes, or lifestyle preferences (62).

#### 2.2.3 Gerontechnology application

The development of intelligent spatial environments has been shown to support older adults in coping more effectively with the functional and lifestyle challenges associated with aging (63). Gerontechnology are designed to leverage emerging technologies and devices to enhance the autonomy, safety, health monitoring, and overall well-being of older adults (63). Prior studies have emphasized that the design of Gerontechnology Applications should prioritize integrated environmental monitoring systems, user–environment interactivity, and intelligent, user-friendly assistive functionalities (64, 65).

The scope of Gerontechnology addressed in this study encompasses multiple design and technological dimensions aimed at enhancing the quality of life and care for older adults, including emergency alert systems (66), fall detection mechanisms, and electronic medical record functions (65). However, there remains a lack of targeted frameworks for integrating age-friendly Gerontechnology with diverse spatial environments. Taken together, prior studies indicate that spatial environments, intelligent technologies, and human-centered, age-friendly design significantly affect older adults’ mental well-being and exposure to structural inequality. Therefore, building on prior literature, this study identifies four dimensions and corresponding evaluation indicators (see Table 1). These dimensions focus on spatial integration from an older adult centered perspective, encompassing Public Spaces, Living Spaces, Age-Friendly Design, and Gerontechnology Application. These dimensions focus on spatial integration from an older adult–centered perspective, encompassing Public Space, Living Space, Gerontechnology Applications, and Age-Friendly Design. The objective is to develop an operational framework that can directly support decision-making and design planning, while systematically offering guidance for the sustainable development and practice of smart cities in aging societies.

**Table 1:** Descriptions of potential dimensions and associated criteria.

Criteria		Description	Reference
Dimension 1: Public Space			
*I*_1_	Cleanliness of public spaces	Public areas are clean and pleasant.	(48, 98–100, 102, 103, 106)
*I*_2_	Quantity and safety of green spaces and outdoor seating	Sufficient green spaces and outdoor seating are provided, well-maintained and safe.	
*I*_3_	Sidewalk maintenance and exclusivity	Sidewalks are well-maintained, free of obstructions, and designated for pedestrian use only.	
*I*_4_	Sidewalk material, width, and leveling	Sidewalks are slip-resistant, wide enough for wheelchairs, and curbs are lowered to road level.	
*I*_5_	Number and safety of pedestrian crossings	Crossings are adequate and safe for individuals with various disabilities, including anti-slip markings, visual and audio cues, and sufficient crossing time.	
*I*_6_	Driver yielding behavior	Drivers yield to pedestrians at intersections and designated crossings.	
*I*_7_	Separation of bicycles from pedestrian paths	Bicycle lanes are separated from pedestrian pathways and sidewalks.	
*I*_8_	Outdoor safety enhancement measures	Adequate street lighting, police patrols, and community education enhance outdoor safety.	
*I*_9_	Service concentration	Services are grouped and accessible.	
*I*_10_	Special customer service arrangements	Separate lines or service counters are available for older adults.	
*I*_11_	Indoor and outdoor signage	Good signage is provided both inside and outside buildings.	
*I*_12_	Indoor and outdoor seating	Sufficient seating is available in and around buildings.	
*I*_13_	Indoor and outdoor restrooms	Adequate restroom facilities are available both inside and outside buildings.	
*I*_14_	Indoor and outdoor accessible features	Facilities include elevators, ramps, handrails, stairways, and non-slip walkways.	
Dimension 2: Living Space			
*I*_15_	Guest room	A spare bedroom is available for guests or as a home office or connection to a guest apartment.	(48, 50, 107–111)
*I*_16_	Building energy rating	Energy performance labels (e.g., CO₂ emissions) are displayed on buildings.	
*I*_17_	Attic insulation	Insulation retains indoor temperature and maintains thermal consistency from ceiling to floor.	
*I*_18_	Thermal water tanks	Water tanks maintain temperature with high resistance to thermal variation.	
*I*_19_	Wall insulation	Includes cavity wall insulation, interior, or exterior insulation.	
*I*_20_	Low-maintenance heating systems	Use of alternatives to solid fuels like wood or coal.	
*I*_21_	Outdoor storage space	Outdoor space is available for storing recyclables, e.g., used appliances.	
*I*_22_	Heating expenditure ratio	Less than 10% of household income is spent on heating.	
*I*_23_	Renewable energy sources	Use of renewable energy sources, such as solar panels.	
*I*_24_	House orientation and daylighting	Good building orientation ensures sunny rooms during the day.	
*I*_25_	Accessible heating controls	Heating systems are easy to control, including temperature settings.	
*I*_26_	Roll-in shower accessibility	Ground floor bathrooms allow for installation of roll-in showers.	
*I*_27_	Bathroom proximity	Bathroom is located adjacent to the main or master bedroom.	
*I*_28_	Indoor natural light	Use of windows, skylights, etc., effectively brings natural light indoors.	
*I*_29_	Comfortable ambient temperature	Living room should maintain 21 °C, and other rooms 18 °C, per WHO recommendations.	
*I*_30_	Soundproofing and quietness	The residence has good sound insulation and a quiet surrounding environment.	
*I*_31_	Color contrast	Color contrast is used in home interiors (e.g., doors, frames, walls) for better visibility.	
Dimension 3: Age-Friendly Design			
*I*_32_	Equitable use	Design is usable by people with diverse abilities.	(52, 90–92, 121, 122)
*I*_33_	Flexibility in use	Accommodates a wide range of preferences and abilities without adjustments.	
*I*_34_	Simple and intuitive use	Design is easy to understand and operate with clear visual instructions.	
*I*_35_	Perceptible feedback	Design provides necessary information effectively through high contrast and auditory cues.	
*I*_36_	Tolerant of error	Layouts minimize risks or consequences of errors and allow for reorientation.	
*I*_37_	Low physical effort	Usable efficiently and comfortably with minimal physical exertion.	
*I*_38_	Ease of access and use	Items are clearly visible and within reach, suitable for various hand types.	
Dimension 4: Gerontechnology Application			
*I*_39_	Mobility monitoring	Continuously monitors the movements of older adults residents within their home.	(45, 65, 66, 88, 89, 113–118)
*I*_40_	Emergency alerts	Sends alerts to family members and care centers during emergencies.	
*I*_41_	Fire detection	Triggers secondary alarms when smoke is detected to ensure safety.	
*I*_42_	Wandering detection and prevention	Detects when the older adults leave home, counts residents and visitors, and monitors night activity.	
*I*_43_	Smart information processing	Analyzes, interprets, and makes decisions based on collected information.	
*I*_44_	Heart rate monitoring	Detects irregular heart activity such as cardiac events.	
*I*_45_	Fall detection	Uses sensors to detect falls in real-time using threshold algorithms.	
*I*_46_	Activity detection	Uses multiple sensors to detect other abnormal activity patterns.	
*I*_47_	Emergency button and touchscreen detection	Detects emergency button and touchscreen signals to request help.	
*I*_48_	Automatic ambulance calling	Uses voice messages to call for ambulance services.	
*I*_49_	Automatic distress messaging	Sends status updates to family and caregivers with requests for help.	
*I*_50_	Telehealth for preliminary diagnosis	Enables video calls between older adults and medical staff for remote diagnosis.	
*I*_51_	Recording of appointments and medication schedules	Displays doctor-scheduled medical appointments and medication plans.	
*I*_52_	Medical consultation via video call	Enables remote video consultation with personal physicians.	
*I*_53_	Social interaction via video call	Allows video calls between older adults and family or friends.	
*I*_54_	Daily and medication schedule tracking	Displays reminders for daily activities and medications set by family members.	
*I*_55_	Touchscreen control panel	Activates all necessary communication systems through an interface, with programmable functionalities.	

## 3 Methodology and data collection

### 3.1 Research methods and procedures

**Figure 1:**
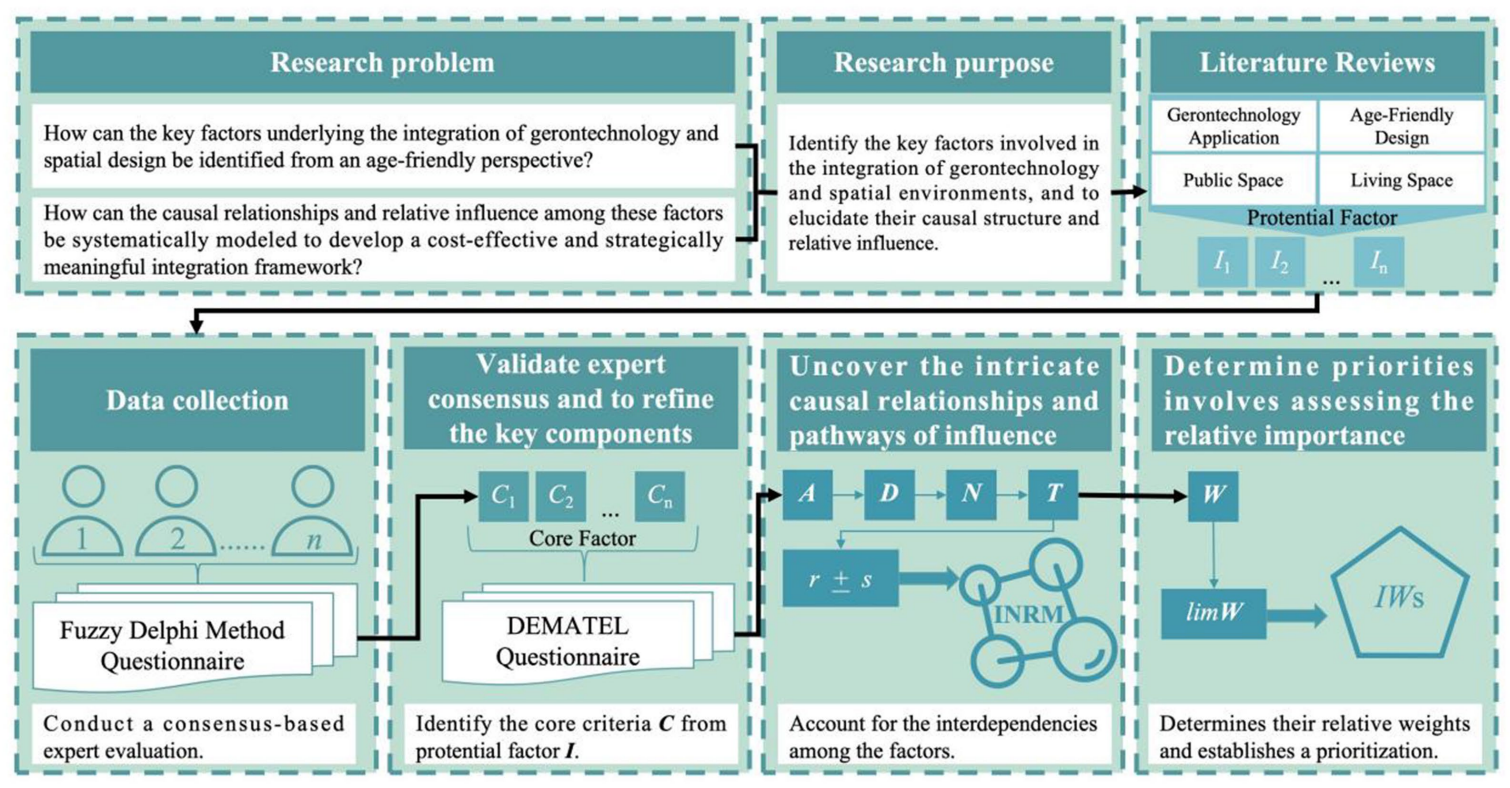
Research procedure.

To address the aforementioned issues, this study employs an expert-based multiple-criteria decision-making (MCDM) approach to develop a systematic age-friendly spatial evaluation framework. Specifically, the FDM is first used to identify the critical factors. Subsequently, the DEMATEL technique is applied to analyze the causal relationships and interactions among these factors. Finally, the DANP is integrated to further derive the relative weights of each factor, as illustrated in Figure 1.

#### 3.1.1 Fuzzy Delphi method

Given the complexity and multidimensionality of smart aging spaces, the FDM is employed to validate expert consensus and to refine the key components of the age-friendly spatial framework. FDM is a MCDM technique designed to manage uncertainty and imprecise information in expert evaluations. Its underlying rationale lies in the integration of fuzzy logic and the Delphi method, enabling structured expert judgment through the use of linguistic variables and fuzzy number processing for problem assessment and decision-making. In early studies, Jeng (67) frequently combined fuzzy theory with the Delphi technique to identify relevant variables and potential evaluation indicators within assessment frameworks. The FDM has since been widely applied in planning and evaluation research across domains such as regional governance, community management, and spatial design (68–71). Compared with the traditional Delphi approach, the fuzzy-enhanced method offers several advantages, including:

1. Fewer iterative survey rounds.
2. Enhanced accuracy in capturing expert knowledge.
3. Effective engagement of domain-relevant experts.
4. Time and cost efficiency in the evaluation process.

The computational procedures are as follows:

Step 1: For each indicator, calculate the minimum values among all experts’ lower bound (*l*) and upper bound (*u*) estimates, and the geometric mean (*m*) of the fuzzy numbers representing expert judgments.

Step 2: Calculate the crisp value *o_i_* using Equation 1.


(1) $$o=o_{i}=\frac{l_{i}+m_{i}+u_{i}}{3},i=1,2,\ldots ,n$$

Step 3: The potential factors are denoted as *I*_i_…I_n_, and the Interquartile Range (IQR) is employed to define a consensus threshold. Indicators meeting this criterion are retained as key factors, labeled (*C*_i_…C_n_).

#### 3.1.2 DEMATEL

In practical decision-making contexts, the factors involved often exhibit highly complex and intertwined relationships, making it difficult for traditional analytical approaches to uncover the underlying causal mechanisms. To address this challenge, Gabus and Fontela (72) introduced the DEMATEL method (73), which was developed to assist researchers and decision-makers in identifying both direct and indirect influences among factors through a systems-thinking perspective, thereby elucidating the causal structure underlying complex problems.

To uncover the intricate causal relationships and pathways of influence within decision-making systems, this study employs the DEMATEL method to identify the core factors involved in the development of age-friendly environments. This approach supports researchers and decision-makers in pinpointing critical issues, thereby enhancing the appropriateness of intelligent spaces for aging populations. DEMATEL effectively differentiates between driving and affected factors and constructs a clear structure of interdependencies (74–79). The analytical procedure is outlined as follows:

Step 1: Calculate the initial average matrix ***A***.

Experts are invited to perform pairwise comparisons among the factors and assign scores on a scale from 0 to 4, representing levels of influence: “no influence,” “low influence,” “moderate influence,” “high influence,” and “very high influence.” The assessments are then aggregated using Equation 2 to compute the resulting initial average matrix ***A***, where ***N*** denotes the total number of *k*th experts involved in the evaluation.


(2) $$\begin{array}{c} A=\frac{1}{N}\sum_{k=1}^{N}A_{\mathrm{ij}}^{k}\\ =[\begin{array}{ccccc} a_{11} & \cdots & a_{1j} & \cdots & a_{1n}\\ \vdots & & \vdots & & \vdots \\ a_{i1} & \cdots & a_{\mathrm{ij}} & \cdots & a_{\text{in}}\\ \vdots & & \vdots & & \vdots \\ a_{n1} & \cdots & a_{\mathrm{mj}} & \cdots & a_{\mathrm{nn}} \end{array}] \end{array}$$

Equation 3 is then applied to assess the degree of consensus among the experts. A confidence level exceeding 95% suggests that the expert evaluations are stable. If the confidence level falls below 95%, Equation 2 must be recalculated, the reliability of the collected data should be verified, and the necessity of including additional experts should be evaluated.


(3) $$\begin{array}{c} \text{Average}\;\mathrm{gap}-\text{ratio}\;\mathrm{on}\;\text{consensus}(\%)=\frac{1}{n(n-1)}\sum_{t=1}^{n}\sum_{j=1}^{n}(|a_{\mathrm{tj}}^{N}-a_{\mathrm{tj}}^{N-1}|a_{\mathrm{tj}}^{N})\\ \times 100\% \end{array}$$

Step 2: Normalization for Constructing the Direct Influence Matrix ***D***.

In this step, the initial average matrix ***A*** is normalized using Equations 4, 5 to construct the direct influence matrix ***D***.


(4) $$s=\max(\max_{1\leq i\leq n}\sum_{j=1}^{n}A_{\mathrm{ij}},\max_{1\leq j\leq n}\sum_{i=1}^{n}A_{\mathrm{ij}})$$

(5) $$D=\frac{A}{s}$$

Step 3: Derivation of the Total Influence Matrix ***T***.

After the convergence of the Markov process, the total influence matrix ***T*** is derived, where ***I*** denotes the identity matrix obtained after an infinite number of influence interactions, as shown in Equation 6.


(6) $$\begin{array}{l} T=N+N^{2}+N^{3}+\cdots +N^{k}\\ =N(I+N+N^{2}+\cdots +N^{k-1})[(I-N){\left(I-N\right)}^{-1}]\\ =N(I-N^{k}){\left(I-N\right)}^{-1}\\ =N{\left(I-N\right)}^{-1},\text{when}\;k\to \infty ,N^{k}={\left[0\right]}_{mxm} \end{array}$$

Step 4: Identification of Interrelationships Among Indicators.

The analysis of interrelationships involves computing four key metrics for each indicator: influence *r*, receptivity *c*, causality *m*, and centrality *p*. Specifically, the influence score quantifies the extent to which a given indicator affects others, and is represented as a vector, as shown in Equation 7.


(7) $$r={\left(r_{i}\right)}_{n\times 1}={\left[\sum_{j-1}^{n}t_{\mathrm{ij}}\right]}_{n\times 1}=(r_{1},\ldots ,r_{i},\ldots ,r_{n})\;\;\;$$

The receptivity score quantifies the extent to which a given indicator is influenced by others. As shown in Equation 8, it is represented in vector *c* form in this study.


(8) $$c={\left(c_{i}\right)}_{n\times 1}=(c_{j}){'}_{1\times n}={\left[\sum_{i-1}^{n}t_{\mathrm{ij}}\right]}_{1\times n}={\left(c_{1},\ldots ,c_{j},\ldots ,c_{n}\right)}^{'}$$

The causality score is defined as the difference between the extent to which an indicator influences others and the extent to which it is influenced. The result is represented as a vector *m*. As shown in Equation 9, a positive *m_i_* value indicates that the indicator acts as a driving factor, while a negative *m_i_* value implies that it functions as a resulting factor.


(9) $$m=m_{i}=r_{i}-c_{i}$$

The centrality score, as shown in Equation 10, is computed as the sum of an indicator’s influence and receptivity scores, and is represented in vector *p*, reflecting the relative importance of the indicator.


(10) $$p=p_{i}=r_{i}+c_{i}$$

Step 5: Construction of the Influence Network-Relation Map (INRM).

Based on the calculated causality (*m*) and centrality (*p*) scores, the INRM is generated to visualize the causal interrelationships and relative importance of the indicators.

Step 6: Construction of the Total Influence Matrix for Criteria and Dimensions.

As shown in Equation 11, the total influence matrix for the criteria $T_{c}={\left[t_{c}^{\mathrm{ij}}\right]}_{n\times n\prime }$ , comprising *n* criteria is established. Similarly, the total influence matrix for the dimensions $T_{D}={\left[t_{D}^{\mathrm{ij}}\right]}_{m\times m\prime }$ , comprising *m* dimensions clustered under the criteria is also constructed.


(11) $$\begin{array}{l} \;D_{1}{\begin{array}{cc} & D \end{array}}_{j}{\begin{array}{cc} & D \end{array}}_{m}\\ \begin{array}{cc} & c_{11}...c_{1n_{1}} \end{array}\cdots c_{j1}...c_{{\mathrm{jn}}_{j}}\cdots c_{m1}...c_{{\mathrm{mn}}_{m}}\\ T_{c}=\begin{array}{c} D_{1}\\ \vdots \\ D_{i}\\ \vdots \\ D_{m} \end{array}\begin{array}{c} c_{11}\\ c_{12}\\ \vdots \\ c_{1n_{1}}\\ \vdots \\ c_{i1}\\ c_{i2}\\ \vdots \\ c_{{\text{in}}_{i}}\\ \vdots \\ c_{m1}\\ c_{m2}\\ \vdots \\ c_{{\mathrm{mn}}_{m}} \end{array}[\begin{array}{ccccc} T_{c}^{11} & \cdots & T_{c}^{1j} & \cdots & T_{c}^{1m}\\ \vdots & & \vdots & & \vdots \\ T_{c}^{i1} & \cdots & T_{c}^{\mathrm{ij}} & \cdots & T_{c}^{\mathrm{im}}\\ \vdots & & \vdots & & \vdots \\ T_{c}^{m1} & \cdots & T_{c}^{\mathrm{mj}} & \cdots & T_{c}^{\mathrm{mm}} \end{array}] \end{array}$$

#### 3.1.3 DANP

When making decisions, decision-makers not only consider the interrelationships among various factors but also attach importance to their prioritization. Determining such priorities involves assessing the relative importance of each factor. To address this need, Saaty (33) proposed the Analytic Network Process (ANP), a systematic comparison approach. ANP extends the Analytic Hierarchy Process (AHP) by accounting for interdependencies among factors, thereby overcoming the AHP’s assumption of factor independence and offering decision-makers a more realistic basis for decision-making (80).

To date, the integration of the ANP with DEMATEL commonly referred to as the DANP method, has been widely applied in various fields, including smart city development (81, 82), improvements in smart homes (83), green open spaces for the older adults (84), public open space design (79), and housing for healthy older adults (85). Therefore, this study further applies the DANP method to account for the interdependence among factors, identify their relative influence weights, and determine the prioritization of factors related to age-friendly spaces, thereby providing a scientific basis for decision-making.

Step 1: Normalization of the Total Influence Matrix ***T****_c_* within Each Dimension.

To derive the unweighted supermatrix, the total influence matrix ***T****_c_* is normalized within each dimension using Equation 12.


(12) $$\begin{array}{l} {\begin{array}{cc} & D \end{array}}_{1}{\begin{array}{cc} & D \end{array}}_{j}{\begin{array}{cc} & D \end{array}}_{n}\\ \begin{array}{cc} & c_{11}...c_{1m_{1}} \end{array}\cdots c_{j1}...c_{{\mathrm{jm}}_{j}}\cdots c_{n1}...c_{{\mathrm{nm}}_{n}}\\ T_{c}^{\alpha }=\begin{array}{c} D_{1}\\ \vdots \\ D_{i}\\ \vdots \\ D_{m} \end{array}\begin{array}{c} c_{11}\\ c_{12}\\ \vdots \\ c_{1m_{1}}\\ \vdots \\ c_{i1}\\ c_{i2}\\ \vdots \\ c_{{\mathrm{im}}_{i}}\\ \vdots \\ c_{n1}\\ c_{n2}\\ \vdots \\ c_{{\mathrm{nm}}_{n}} \end{array}[\begin{array}{ccccc} T_{c}^{\alpha 11} & \cdots & T_{c}^{\alpha 1j} & \cdots & T_{c}^{\alpha 1n}\\ \vdots & & \vdots & & \vdots \\ T_{c}^{\mathrm{\alpha i}1} & \cdots & T_{c}^{\mathrm{\alpha ij}} & \cdots & T_{c}^{\mathrm{\alpha in}}\\ \vdots & & \vdots & & \vdots \\ T_{c}^{\mathrm{\alpha n}1} & \cdots & T_{c}^{\mathrm{\alpha nj}} & \cdots & T_{c}^{\mathrm{\alpha nn}} \end{array}] \end{array}$$

The total influence matrix normalized by dimension is denoted as $T_{c}^{\alpha }$ . As illustrated by dimension $T_{c}^{\alpha 11}$ , this matrix $T_{c}^{\mathrm{\alpha mm}}$ can be derived using Equations 13, 14.


(13) $$t_{i}^{11}=\sum_{j=1}^{m_{1}}t_{\mathrm{ij}}^{11},i=1,2,\ldots ,m_{1}$$

(14) $$\begin{array}{c} T_{c}^{\alpha 11}=[\begin{array}{ccccc} \frac{t_{c11}^{11}}{t_{c1}^{11}} & \cdots & \frac{t_{c1j}^{11}}{t_{c1}^{11}} & \cdots & \frac{t_{c1m_{1}}^{11}}{t_{c1}^{11}}\\ \vdots & & \vdots & & \vdots \\ \frac{t_{\mathrm{ci}1}^{11}}{t_{\mathrm{ci}}^{11}} & \cdots & \frac{t_{\mathrm{cij}}^{11}}{t_{\mathrm{ci}}^{11}} & \cdots & \frac{t_{\mathrm{ci}m_{1}}^{11}}{t_{\mathrm{ci}}^{11}}\\ \vdots & & \vdots & & \vdots \\ \frac{t_{cm_{1}1}^{11}}{t_{cm_{1}}^{11}} & \cdots & \frac{t_{cm_{1j}}^{11}}{t_{cm_{1}}^{11}} & \cdots & \frac{t_{cm_{1}m_{1}}^{11}}{t_{cm_{1}}^{11}} \end{array}]\\ =[\begin{array}{ccccc} t_{c11}^{\alpha 11} & \cdots & t_{c1j}^{\alpha 11} & \cdots & t_{c1m_{1}}^{\alpha 11}\\ \vdots & & \vdots & & \vdots \\ t_{\mathrm{ci}1}^{\alpha 11} & \cdots & t_{\mathrm{cij}}^{\alpha 11} & \cdots & t_{\mathrm{ci}m_{1}}^{\alpha 11}\\ \vdots & & \vdots & & \vdots \\ t_{cm_{1}1}^{\alpha 11} & \cdots & t_{cm_{1}j}^{\alpha 11} & \cdots & t_{cm_{1}m_{1}}^{\alpha 11} \end{array}] \end{array}$$

Step 2: Construction of the Unweighted Supermatrix *W^α^*.

Following the pairwise comparison mechanism of ANP, the total influence matrix normalized by dimension is transposed using Equation 15 to construct the unweighted supermatrix *W^α^*, denoted as $W^{\alpha }={\left(T_{c}^{\alpha }\right)}^{'}$ .


(15) $$\begin{array}{l} {\begin{array}{cc} & D \end{array}}_{1}{\begin{array}{cc} & D \end{array}}_{i}{\begin{array}{cc} & D \end{array}}_{m}\\ \begin{array}{cc} & c_{11}...c_{1m_{1}} \end{array}\cdots c_{i1}...c_{{\mathrm{im}}_{j}}\cdots c_{n1}...c_{{\mathrm{nm}}_{n}}\\ W^{\alpha }={\left(T_{c}^{\alpha }\right)}^{'}=\begin{array}{c} D_{1}\\ \vdots \\ D_{j}\\ \vdots \\ D_{n} \end{array}\begin{array}{c} c_{11}\\ c_{12}\\ \vdots \\ c_{1m_{1}}\\ \vdots \\ c_{i1}\\ c_{i2}\\ \vdots \\ c_{{\mathrm{jm}}_{i}}\\ \vdots \\ c_{n1}\\ c_{n2}\\ \vdots \\ c_{{\mathrm{nm}}_{n}} \end{array}[\begin{array}{ccccc} W^{11} & \cdots & W^{i1} & \cdots & W^{n1}\\ \vdots & & \vdots & & \vdots \\ W^{1j} & \cdots & W^{\mathrm{ij}} & \cdots & W^{\mathrm{nj}}\\ \vdots & & \vdots & & \vdots \\ W^{1n} & \cdots & W^{\text{in}} & \cdots & W^{\mathrm{nn}} \end{array}] \end{array}$$

Step 3: Calculation of the Total Influence Matrix Normalized by Dimension ***T****_D_*.

Using Equations 16, 17, the total influence matrix ***T****_D_* is divided by the dimensional sum matrix *d_i_*, which yields the dimension-normalized total influence matrix $T_{D}^{\alpha }$ .


(16) $$d_{i}=\sum_{j=1}^{m}t_{\mathrm{ij}},i=1,2,\ldots ,m$$

(17) $$\begin{array}{c} T_{D}^{\alpha }=[\begin{array}{ccccc} \frac{t_{11}}{d_{1}} & \cdots & \frac{t_{1j}}{d_{1}} & \cdots & \frac{t_{1m}}{d_{1}}\\ \vdots & & \vdots & & \vdots \\ \frac{t_{i1}}{d_{i}} & \cdots & \frac{t_{\mathrm{ij}}}{d_{i}} & \cdots & \frac{t_{\mathrm{im}}}{d_{i}}\\ \vdots & & \vdots & & \vdots \\ \frac{t_{m1}}{d_{m}} & \cdots & \frac{t_{\mathrm{mj}}}{d_{m}} & \cdots & \frac{t_{\mathrm{mm}}}{d_{m}} \end{array}]\\ =[\begin{array}{ccccc} t_{11}^{\mathrm{\alpha D}} & \cdots & t_{1j}^{\mathrm{\alpha D}} & \cdots & t_{1m_{1}}^{\mathrm{\alpha D}}\\ \vdots & & \vdots & & \vdots \\ t_{i1}^{\mathrm{\alpha D}} & \cdots & t_{\mathrm{ij}}^{\mathrm{\alpha D}} & \cdots & t_{\mathrm{im}}^{\mathrm{\alpha D}}\\ \vdots & & \vdots & & \vdots \\ t_{m1}^{\mathrm{\alpha D}} & \cdots & t_{\mathrm{mj}}^{\mathrm{\alpha D}} & \cdots & t_{\mathrm{mm}}^{\mathrm{\alpha D}} \end{array}] \end{array}$$

Step 4: Calculation of the Weighted Supermatrix ***W***.

Using Equation 18, the dimension-normalized total influence matrix $T_{D}^{\alpha }$ is multiplied by the unweighted supermatrix *W^α^* to derive the weighted supermatrix ***W***.


(18) $$W=T_{D}^{\alpha }W^{\alpha }=[\begin{array}{ccccc} t_{11}^{\mathrm{\alpha D}}\times W^{11} & \cdots & t_{i1}^{\mathrm{\alpha D}}\times W^{i1} & \cdots & t_{m1}^{\mathrm{\alpha D}}\times W^{m1}\\ \vdots & & \vdots & & \vdots \\ t_{1j}^{\mathrm{\alpha D}}\times W^{1j} & \cdots & t_{\mathrm{ij}}^{\mathrm{\alpha D}}\times W^{\mathrm{ij}} & \cdots & t_{\mathrm{mj}}^{\mathrm{\alpha D}}\times W^{\mathrm{mj}}\\ \vdots & & \vdots & & \vdots \\ t_{1m}^{\mathrm{\alpha D}}\times W^{1m} & \cdots & t_{\mathrm{im}}^{\mathrm{\alpha D}}\times W^{\mathrm{im}} & \cdots & t_{\mathrm{mm}}^{\mathrm{\alpha D}}\times W^{\mathrm{mm}} \end{array}]$$

Step 5: Computation of the Limiting Weighted Supermatrix ***W*** and Weight Derivation.

According to Equation 19, the weighted supermatrix ***W*** is iteratively raised to the power of *z*th until convergence is reached, resulting in the limiting weighted supermatrix. The local weight of each dimension is obtained by summing the weights of all its associated criteria. Subsequently, the global weight of each criterion is divided by the local weight of its corresponding dimension to obtain the criterion’s local weight.


(19) $$\lim_{z\to \infty }{\left(w^{\infty }\right)}^{z}$$

### 3.2 Data collection

To obtain reliable and context-relevant expert input, this study invited participants from diverse domains, including government, industry, and academia, thereby ensuring a broad range of expertise and disciplinary perspectives (see Table 2). Experts were invited to participate in this study through online channels (SMS and Google Forms) To minimize dominance bias, each expert was required to complete the questionnaire independently. All expert information and questionnaire data collected during the process were accessible solely to the researchers for analysis. Data collection was carried out in two rounds:

**Table 2:** Experts background.

No.	Gender	Age	Education	Title	Organization	Seniority (years)
Round 1: FDM						
Exp_1_	M	41–50	PhD	Project Manager	Government Department	>10
Exp_2_	F	41–50	PhD	Marketing Director	Marketing Firm	>10
Exp_3_	M	51–60	PhD	Chief Executive Officer, Professor	Intelligent Technology Company; University	>10
Exp_4_	M	>60	Bachelor	Customer Service Officer	Management Service Company	>10
Exp_5_	M	>60	Bachelor	Manager	Building Management Company	>10
Exp_6_	M	31–40	PhD	Professional Leader	Government Planning Department	3–5
Round 2: DANP						
Exp_1_	M	41–50	Master	Project Manager	Government Department	>10
Exp_2_	M	51–60	Master	Business Unit	Internet Information Organization	>10
Exp_3_	F	51–60	Master	Administrator	Government Department	>10
Exp_4_	M	25–30	PhD	Researcher	Public Policy Institute	3–5
Exp_5_	M	41–50	Master	Vice President	Intelligent Technology Enterprise	>10
Exp_6_	F	25–30	PhD	Research Assistant	School of Architecture	3–5
Exp_7_	M	25–30	Master	Design Assistant	Research Firm	3–5
Exp_8_	F	31–40	PhD	Researcher	School of Humanities and Social Sciences	3–5
Exp_9_	M	25–30	Master	Researcher	Research Institute	3–5
Exp_10_	M	41–50	Master	Business Director	Building Supplies Company	>10
Exp_11_	M	25–30	Master	Research Assistant	School of Architecture	3–5
Exp_12_	M	31–40	PhD	Researcher	Research Institute	3–5

The first round survey of this study was conducted in March 2025, during which six experts with practical experience were invited to complete the FDM questionnaire (Appendix 1). In the questionnaire, experts were asked to assess the importance of 55 potential factors identified in prior literature, with the aim of identifying the key factors underlying the integration of Gerontechnology and spatial contexts. Each expert held a senior management position and possessed either more than ten years of professional experience or a doctoral degree. Their areas of expertise included government planning, higher education, Gerontechnology, and consumer services, thereby ensuring their ability to assess key factors from both macro-level policy and industry practice perspectives.

The second round survey of this study was conducted between April and May 2025. To improve the robustness and objectivity of the findings, experts from both academia and industry were invited to complete the DEMATEL questionnaire (Appendix 2). Experts were asked to conduct pairwise comparisons of the key factors identified in the first round, in order to further elucidate their interrelationships and relative importance. After collecting responses from 12 experts, the researchers synthesized the results and assessed their consistency using Equation 3. Panel consensus was considered stable if the consistency level exceeded 95%, in which case no additional experts were required (79, 86). In the second round, the consensus index for all 12 experts exceeded 95% (99.75, 99.51, 99.46, 99.47%). These results indicate that the panel demonstrated reliable representativeness and consensus, thereby concluding the data collection process.

Overall, the 12 experts invited in this round comprised both early career scholars and senior practitioners, and most of them held master’s or doctoral degrees. Their affiliations spanned government agencies, research institutions, technology firms, and the construction sector, providing a combination of extensive research insights and practical experience that enhanced the robustness and objectivity of the study’s findings. Notably, several experts specialized in Gerontechnology Applications, the humanities and social sciences, architecture, and public policy. Their interdisciplinary expertise enabled a comprehensive evaluation of the interdependencies among factors, thereby reinforcing the study’s systematic understanding of smart age-friendly space planning.

## 4 Results and discussions

### 4.1 Key criteria by FDM

Based on the evaluations of six experts using the FDM and the inter-quartile range (IQR) analysis, the threshold for identifying key factors was determined to be 4.44. Consequently, 15 key factors (*C*_1_–*C*_15_) were selected from an initial pool of 55 potential items (*I*_1_–*I*_55_) and classified into four dimensions: Public Space (***D***_1_), Living Space (***D***_2_), Age-Friendly Design (***D***_3_), and Gerontechnology Application (***D***_4_). Specifically, Public Space dimension included Pavement maintenance and dedication (*C*_1_), Pavement material, width and level (*C*_2_), Separation of bicycles from pavement (*C*_3_) and Outdoor safety enhancement measures (*C*_4_); While Living Space dimension emphasized Barrier-free access to and within buildings (*C*_5_), Environmental design for daylight access and natural ventilation (*C*_6_ to *C*_8_), and Comfortable ambient temperature (*C*_9_). The Age-Friendly Design dimension incorporates Fault-tolerant spatial configuration (*C*_10_), which minimizes risks arising from unintentional user actions. Lastly, the Gerontechnology Application dimension focuses on the application of Gerontechnology for emergency and daily support, including emergency notification dispatch (*C*_11_), fire and fall detection (*C*_12_ to *C*_13_), Automatic transmission of distress messages (*C*_14_), and Appointment and medication schedule reminders (*C*_15_). The results are shown as Table 3 and the detailed description of core framework are shown in Table 4.

**Table 3:** Classification of key criteria across core dimensions.

Code	*l*	*m*	*u*	*o*	Decision (>4.44)	No.
*I*_1_	3.00	3.96	5.00	3.99	Delete	
*I*_2_	3.00	3.91	5.00	3.97	Delete	
*I*_3_	4.00	4.47	5.00	4.49	KEEP	*C*_1_
*I*_4_	4.00	4.31	5.00	4.44	KEEP	*C*_2_
*I*_5_	3.00	3.96	5.00	3.99	Delete	
*I*_6_	2.00	3.70	5.00	3.57	Delete	
*I*_7_	4.00	4.47	5.00	4.49	KEEP	*C*_3_
*I*_8_	4.00	4.31	5.00	4.44	KEEP	*C*_4_
*I*_9_	4.00	4.00	4.00	4.00	Delete	
*I*_10_	4.00	4.15	5.00	4.38	Delete	
*I*_11_	4.00	4.00	4.00	4.00	Delete	
*I*_12_	3.00	3.81	4.00	3.60	Delete	
*I*_13_	3.00	4.11	5.00	4.04	Delete	
*I*_14_	4.00	4.47	5.00	4.49	KEEP	*C*_5_
*I*_15_	2.00	3.24	4.00	3.08	Delete	
*I*_16_	1.00	2.40	4.00	2.47	Delete	
*I*_17_	2.00	3.17	4.00	3.06	Delete	
*I*_18_	2.00	3.40	4.00	3.13	Delete	
*I*_19_	2.00	3.17	4.00	3.06	Delete	
*I*_20_	3.00	3.81	4.00	3.60	Delete	
*I*_21_	2.00	3.24	4.00	3.08	Delete	
*I*_22_	1.00	2.70	4.00	2.57	Delete	
*I*_23_	1.00	2.67	5.00	2.89	Delete	
*I*_24_	4.00	4.47	5.00	4.49	KEEP	*C*_6_
*I*_25_	3.00	4.11	5.00	4.04	Delete	
*I*_26_	4.00	4.64	5.00	4.55	KEEP	*C*_7_
*I*_27_	3.00	4.11	5.00	4.04	Delete	
*I*_28_	4.00	4.31	5.00	4.44	KEEP	*C*_8_
*I*_29_	4.00	4.47	5.00	4.49	KEEP	*C*_9_
*I*_30_	3.00	4.11	5.00	4.04	Delete	
*I*_31_	2.00	3.09	4.00	3.03	Delete	
*I*_32_	3.00	3.63	4.00	3.54	Delete	
*I*_33_	2.00	3.40	4.00	3.13	Delete	
*I*_34_	1.00	3.42	5.00	3.14	Delete	
*I*_35_	3.00	4.26	5.00	4.09	Delete	
*I*_36_	4.00	4.31	5.00	4.44	KEEP	*C*_10_
*I*_37_	3.00	3.96	5.00	3.99	Delete	
*I*_38_	1.00	3.30	5.00	3.10	Delete	
*I*_39_	3.00	4.11	5.00	4.04	Delete	
*I*_40_	4.00	4.64	5.00	4.55	KEEP	*C*_11_
*I*_41_	4.00	4.64	5.00	4.55	KEEP	*C*_12_
*I*_42_	3.00	3.96	5.00	3.99	Delete	
*I*_43_	2.00	3.66	5.00	3.55	Delete	
*I*_44_	3.00	3.91	5.00	3.97	Delete	
*I*_45_	4.00	4.64	5.00	4.55	KEEP	*C*_13_
*I*_46_	3.00	4.11	5.00	4.04	Delete	
*I*_47_	3.00	4.26	5.00	4.09	Delete	
*I*_48_	3.00	4.26	5.00	4.09	Delete	
*I*_49_	4.00	4.64	5.00	4.55	KEEP	*C*_14_
*I*_50_	3.00	4.11	5.00	4.04	Delete	
*I*_51_	4.00	4.47	5.00	4.49	KEEP	*C*_15_
*I*_52_	2.00	3.30	5.00	3.43	Delete	
*I*_53_	2.00	3.40	4.00	3.13	Delete	
*I*_54_	2.00	3.70	5.00	3.57	Delete	
*I*_55_	1.00	3.30	5.00	3.10	Delete	

The threshold values are Q1: 3.29, Q2:4.00, and Q3:4.44 (Inter quartile range, IQR).

**Table 4:** Descriptions of core dimensions and associated criteria.

Dimension/criteria		Description	Reference
***D***_1_	*Public Space*	Public Spaces play a crucial role in fostering social inclusion, mobility, and safety for older adults. The design principles under this dimension emphasize accessibility, visibility, and environmental predictability.	
*C*_1_	Pavement maintenance and dedication	Pavements should be regularly maintained, free of obstructions, and designated solely for pedestrian use. This ensures safe mobility and reduces the risk of accidents such as trips and falls.	(48, 106)
*C*_2_	Pavement material, width and level	Walking surfaces should be constructed with non-slip materials and maintain adequate width to accommodate assistive devices like wheelchairs or walkers. Level surfaces with minimized height differences contribute significantly to ease of navigation for mobility-impaired individuals.	(48, 98, 99)
*C*_3_	Separation of bicycles from pavement	Clearly demarcated zones that segregate pedestrian walkways from cycling lanes or vehicular traffic reduce collision risk and foster a sense of safety for older users in shared urban spaces.	(48, 100)
*C*_4_	Outdoor safety enhancement measures	Features such as adequate street lighting, surveillance systems, wayfinding signage, and the presence of community patrols create a secure outdoor environment, especially during evening hours or in isolated areas.	(48, 106)
*C*_5_	Barrier-free access to and within buildings	Buildings should incorporate universally accessible features such as ramps, elevators, tactile guides, handrails, and accessible toilets, ensuring seamless entry, exit, and interior navigation for older adults, thereby supporting independent living.	(48, 102, 103)
***D*** *_2_*	*Living Space*	The Living Space dimension focuses on the home environment, which is central to aging in place strategies. It prioritizes adaptability, environmental comfort, and future proofing for functional decline.	
*C*_6_	Orientation and lighting	Proper house orientation enables natural daylighting throughout the day, contributing to circadian rhythm regulation, improved mood, and enhanced visual comfort, especially for those with vision impairment.	(48, 50, 107)
*C*_7_	Roll-in shower adaptability	Bathrooms should be designed with sufficient space and layout flexibility to accommodate future installation of roll-in showers or transfer equipment, facilitating a seamless transition to accessible hygiene routines as mobility declines.	(48, 50, 109, 110)
*C*_8_	Indoor natural light access	The use of architectural elements such as large windows and skylights increases natural light penetration, reducing the need for artificial lighting, and supporting visual clarity and spatial awareness in older users.	(48, 50, 107, 108)
*C*_9_	Comfortable ambient temperature	Indoor temperature should be maintained within the World Health Organization’s recommended range (e.g., ≥18 °C in occupied rooms). Proper thermal conditions reduce health risks, particularly cardiovascular complications, and promote residential comfort.	(48, 50, 111)
***D***_3_	*Age-Friendly Design*	This dimension emphasizes spatial features that align with older adults’ behavioral patterns, cognitive needs, and risk profiles. It supports autonomy while minimizing accident potential.	
C_10_	Fault-tolerant spatial configuration	Environments should be designed with error tolerance in mind, e.g., wide corridors, looped circulation, intuitive layouts, and clear signage, to allow users to reorient themselves easily and return safely to common areas, even in cases of disorientation or cognitive decline.	(52, 90–92, 121, 122)
***D***_4_	*Gerontechnology Application*	Spaces integrate gerontechnological solutions to enhance health monitoring, emergency responsiveness, and daily life assistance. This dimension reflects a transition from passive environments to active care systems.	
*C*_11_	Emergency notification dispatch	In the event of emergencies, intelligent systems should automatically notify caregivers or family members, ensuring a rapid response and reducing the severity of medical complications due to delays.	(45, 65, 66, 113, 118)
*C*_12_	Fire detection	Smoke or heat sensors should be integrated into the home infrastructure. Upon detecting fire-related incidents, systems must activate alarms and initiate pre-set emergency communication protocols to alert both the resident and external responders.	(66, 88, 118)
*C*_13_	Fall detection	Wearable or ambient sensors using triaxial accelerometers and 3D motion analysis should be employed to detect falls in real-time. Such systems are vital in providing prompt intervention, particularly for older adults living alone.	(65, 88, 114–116)
*C*_14_	Automatic transmission of distress messages	Systems should be capable of automatically sending alerts, including location and status updates, to designated caregivers, offering essential communication support when the older adults are unable to act independently.	(45, 65, 113, 117)
*C*_15_	Appointment and medication schedule reminders	Smart displays or voice assistants should provide timely reminders about medical appointments and medication schedules set by healthcare providers, thereby enhancing treatment adherence and reducing the burden on caregivers.	(65, 89, 114)

### 4.2 Analysis of causal relationships among core criteria through the DEMATEL approach

**Figure 2:**
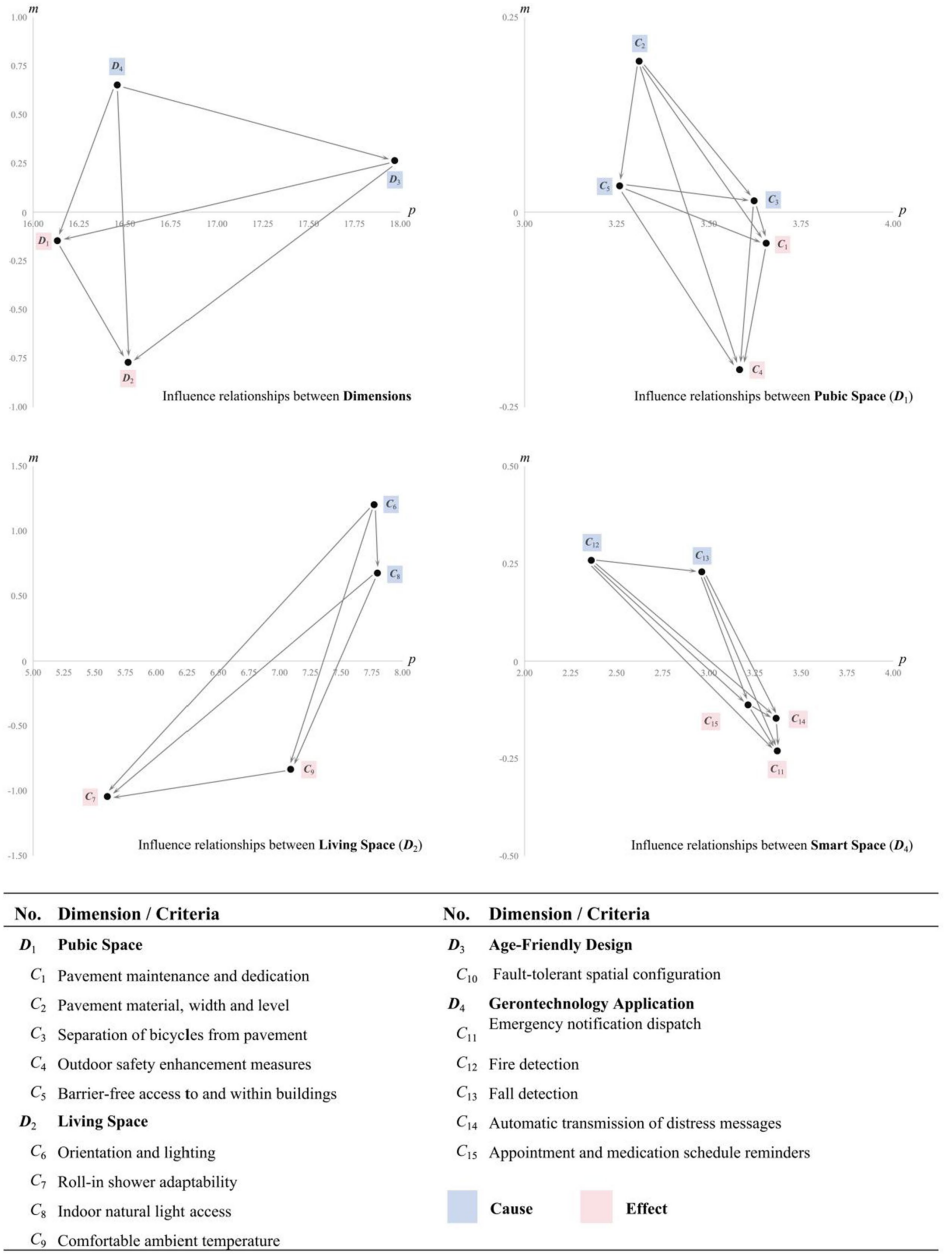
Influence network relationship map (INRM).

Through the application of the DEMATEL method, this study further clarifies the core criteria and the interrelationships among them in the context of constructing Age-Friendly and Gerontechnology Applications. A consensus analysis was conducted based on Equation 3, and the resulting consensus indices 99.75, 99.51, 99.46, and 99.47%. All exceeded the 95% threshold, indicating that the expert panel was both highly representative and demonstrated a strong level of agreement (87). Furthermore, based on the average direct influence matrix derived from expert evaluations, the influence relationships between the dimensions and the criteria were calculated individually (see Table 5), leading to the construction of the total influence matrix (Table 6). Subsequently, this matrix was employed to determine the strength and direction of influence among the dimensions and criteria (Table 7), which were then illustrated using the INRM (Figure 2). This figure provides a visual representation of the causal relationships and the corresponding levels of influence among the identified criteria.

**Table 5:** The average direct-influence relation matrix ***A***.

Dimension	*D*_1_	*D*_2_	*D*_3_	*D*_4_	
***D***_1_	0.00	2.50	3.33	2.58	
***D***_2_	2.25	0.00	3.25	2.75	
***D***_3_	3.42	3.67	0.00	3.00	
***D***_4_	3.00	3.17	3.08	0.00	
Criteria					
***D***_1_	*C*_1_	*C*_2_	*C*_3_	*C*_4_	*C*_5_
*C*_1_	0.00	2.75	3.58	3.33	2.83
*C*_2_	3.25	0.00	2.67	3.33	2.83
*C*_3_	3.67	2.67	0.00	3.67	2.58
*C*_4_	2.92	2.58	3.33	0.00	2.75
*C*_5_	3.00	2.58	2.83	2.83	0.00
***D***_2_	*C*_6_	*C*_7_	*C*_8_	*C*_9_	
*C*_6_	0.00	1.33	3.42	2.67	
*C*_7_	1.25	0.00	1.00	1.17	
*C*_8_	2.25	2.25	0.00	2.83	
*C*_9_	1.75	1.75	1.50	0.00	
***D***_4_	*C*_11_	*C*_12_	*C*_13_	*C*_14_	*C*_15_
*C*_11_	0.00	2.42	2.83	3.50	2.83
*C*_12_	2.75	0.00	1.25	3.08	2.42
*C*_13_	3.67	1.00	0.00	3.33	3.67
*C*_14_	3.50	2.42	2.58	0.00	3.42
*C*_15_	3.58	1.50	3.00	3.25	0.00

**Table 6:** The total influence matrix ***T***.

Dimension	*D*_1_	*D*_2_	*D*_3_	*D*_4_	
***D***_1_	1.78	2.09	2.19	1.93	
***D***_2_	1.94	1.87	2.15	1.92	
***D***_3_	2.28	2.42	2.21	2.20	
***D***_4_	2.13	2.26	2.31	1.85	
Criteria					
***D***_1_	*C*_1_	*C*_2_	*C*_3_	*C*_4_	*C*_5_
*C*_1_	0.23	0.36	0.42	0.43	0.37
*C*_2_	0.41	0.18	0.38	0.42	0.36
*C*_3_	0.43	0.35	0.23	0.44	0.36
*C*_4_	0.39	0.33	0.40	0.22	0.35
*C*_5_	0.38	0.33	0.37	0.38	0.18
***D***_2_	*C*_6_	*C*_7_	*C*_8_	*C*_9_	
*C*_6_	0.87	1.03	1.27	1.32	
*C*_7_	0.58	0.44	0.60	0.66	
*C*_8_	1.04	1.05	0.88	1.26	
*C*_9_	0.79	0.80	0.82	0.72	
***D***_4_	*C*_11_	*C*_12_	*C*_13_	*C*_14_	*C*_15_
*C*_11_	0.22	0.26	0.32	0.40	0.36
*C*_12_	0.34	0.10	0.22	0.35	0.30
*C*_13_	0.42	0.20	0.17	0.40	0.40
*C*_14_	0.41	0.27	0.32	0.22	0.39
*C*_15_	0.41	0.22	0.33	0.39	0.20

**Table 7:** Strength of influence and causality between evaluation.

	*r*	*s*	*m*	*p*
***D***_1_	7.99	8.14	16.13	−0.15
*C*_1_	1.81	1.85	3.66	−0.04
*C*_2_	1.75	1.56	3.31	0.19
*C*_3_	1.82	1.80	3.62	0.01
*C*_4_	1.69	1.89	3.58	−0.20
*C*_5_	1.65	1.61	3.26	0.03
***D***_2_	7.87	8.64	16.52	−0.77
*C*_6_	4.49	3.28	7.77	1.20
*C*_7_	2.28	3.32	5.60	−1.04
*C*_8_	4.24	3.56	7.80	0.68
*C*_9_	3.13	3.96	7.09	−0.83
***D***_3_	9.12	8.85	17.97	0.26
*C*_10_	9.12	8.85	17.97	0.26
***D***_4_	8.56	7.90	16.46	0.65
*C*_11_	1.57	1.80	3.37	−0.23
*C*_12_	1.31	1.05	2.36	0.26
*C*_13_	1.60	1.37	2.96	0.23
*C*_14_	1.61	1.76	3.36	−0.15
*C*_15_	1.55	1.66	3.21	−0.11

The results derived from the INRM indicated that among the four dimensions, Gerontechnology Application (***D***_4_) exhibits the highest overall influence, followed by Age-Friendly Design (***D***_3_), Public Space (***D***_1_), and Living Space (***D***_2_), with a descending order of ***D***_4_ > ***D***_3_ > ***D***_1_ > ***D***_2_. Within the causal structure, ***D***_4_ and ***D***_3_ are identified as Cause dimensions, that exert significant influence within the system, whereas ***D***_1_ and ***D***_2_ are classified as Effect dimensions, being more strongly influenced by the others.

At the criteria level, the INRM reveals the internal causal relationships within each dimension. In Public Space dimension (***D***_1_), Pavement material, width, and level (*C*_2_) exerts the strongest influence, resulting in the ranking order: *C*_2_ > *C*_5_ > *C*_3_ > *C*_1_ > *C*_4_. Among these, *C*_2_, *C*_5_ and *C*_3_ are identified as Cause criteria, while *C*_1_ and *C*_4_ are classified as Effect criteria. In the Living Space dimension (***D***_2_), Orientation and lighting (*C*_6_) is the emerges as the most influential factor, followed by *C*_6_ > *C*_8_ > *C*_9_ > *C*_7_. Both *C*_6_ and *C*_8_ are identified as Cause criteria. For Age-Friendly Design dimension (***D***_3_), although it includes only one criteria (Fault-tolerant spatial configuration, *C*_10_), it should not be underestimated as it is identified as the Cause dimensions. Within the Gerontechnology Application dimension (***D***_4_), Fire detection (*C*_12_) demonstrates the highest level of influence among all criteria in the dimension, resulting in the ranking order: *C*_12_ > *C*_13_ > *C*_15_ > *C*_14_ > *C*_11_.

### 4.3 Determination of criteria weights using the DANP method

As shown in Table 8, the Unweighted Supermatrix *W^α^* captures the initial interrelationships among all criteria, prior to normalization and convergence within the DANP procedure. This matrix serves as the computational basis for deriving the final priority weights. Building upon this structure, the DANP analysis quantifies the relative importance of each dimension and its associated criteria (Table 9).

**Table 8:** Unweighted supermatrix *W^α^*.

	*C*_1_	*C*_2_	*C*_3_	*C*_4_	*C*_5_	*C*_6_	*C*_7_	*C*_8_	*C*_9_	*C*_10_	*C*_11_	*C*_12_	*C*_13_	*C*_14_	*C*_15_
*C*_1_	0.13	0.23	0.24	0.23	0.23	0.00	0.00	0.00	0.00	0.00	0.00	0.00	0.00	0.00	0.00
*C*_2_	0.20	0.11	0.19	0.20	0.20	0.00	0.00	0.00	0.00	0.00	0.00	0.00	0.00	0.00	0.00
*C*_3_	0.24	0.22	0.13	0.24	0.23	0.00	0.00	0.00	0.00	0.00	0.00	0.00	0.00	0.00	0.00
*C*_4_	0.24	0.24	0.24	0.13	0.23	0.00	0.00	0.00	0.00	0.00	0.00	0.00	0.00	0.00	0.00
*C*_5_	0.20	0.21	0.20	0.21	0.11	0.00	0.00	0.00	0.00	0.00	0.00	0.00	0.00	0.00	0.00
*C*_6_	0.00	0.00	0.00	0.00	0.00	0.19	0.25	0.25	0.25	0.00	0.00	0.00	0.00	0.00	0.00
*C*_7_	0.00	0.00	0.00	0.00	0.00	0.23	0.19	0.25	0.25	0.00	0.00	0.00	0.00	0.00	0.00
*C*_8_	0.00	0.00	0.00	0.00	0.00	0.28	0.26	0.21	0.26	0.00	0.00	0.00	0.00	0.00	0.00
*C*_9_	0.00	0.00	0.00	0.00	0.00	0.29	0.29	0.30	0.23	0.00	0.00	0.00	0.00	0.00	0.00
*C*_10_	0.00	0.00	0.00	0.00	0.00	0.00	0.00	0.00	0.00	1.00	0.00	0.00	0.00	0.00	0.00
*C*_11_	0.00	0.00	0.00	0.00	0.00	0.00	0.00	0.00	0.00	0.00	0.14	0.26	0.27	0.26	0.27
*C*_12_	0.00	0.00	0.00	0.00	0.00	0.00	0.00	0.00	0.00	0.00	0.17	0.08	0.12	0.17	0.14
*C*_13_	0.00	0.00	0.00	0.00	0.00	0.00	0.00	0.00	0.00	0.00	0.21	0.17	0.11	0.20	0.22
*C*_14_	0.00	0.00	0.00	0.00	0.00	0.00	0.00	0.00	0.00	0.00	0.26	0.26	0.25	0.13	0.25
*C*_15_	0.00	0.00	0.00	0.00	0.00	0.00	0.00	0.00	0.00	0.00	0.23	0.23	0.25	0.24	0.13

**Table 9:** Calculated local and global weights for all criteria.

Dimensions	Local weight	Criteria	Local weight	Global weight	Rank
***D***_1_	24.25%	*C*_1_	21.15%	5.13%	9
		*C*_2_	18.07%	4.38%	13
		*C*_3_	20.75%	5.03%	11
		*C*_4_	21.49%	5.21%	8
		*C*_5_	18.53%	4.49%	12
***D***_2_	25.71%	*C*_6_	23.66%	6.08%	4
		*C*_7_	23.14%	5.95%	5
		*C*_8_	25.26%	6.49%	3
		*C*_9_	27.94%	7.18%	2
***D***_3_	26.42%	*C*_10_	100%	26.42%	1
***D***_4_	23.62%	*C*_11_	23.25%	5.49%	6
		*C*_12_	14.12%	3.33%	15
		*C*_13_	18.26%	4.31%	14
		*C*_14_	22.77%	5.38%	7
		*C*_15_	21.59%	5.10%	10

Among the four primary dimensions, Age-Friendly Design (***D***_3_) attained the highest local weight (26.42%), followed by Living Space (***D***_2_) (25.71%), Public Space (***D***_1_) (24.25%), and Gerontechnology Application (***D***_4_) (23.62%). At the criteria level, Fault-tolerant spatial configuration (*C*_10_) emerged as the most influential criteria overall, with a global weight of 26.42%, ranking first among all criteria. The complete ranking of criteria based on global weight is as follows: C10 (26.42%) > C9 (7.18%) > C8 (6.49%) > C6 (6.08%) > *C*_7_ (5.95%) > *C*_11_ (5.49%) > *C*_14_ (5.38%) > *C*_4_ (5.21%) > *C*_1_ (5.13%) > *C*_15_ (5.10%) > *C*_3_ (5.03%) > *C*_5_ (4.49%) > *C*_2_ (4.38%) > *C*_13_ (4.31%) > *C*_12_ (3.33%).

### 4.4 Discussion

This study employed a hybrid MCDM model combining FDM and DANP to systematically identify and evaluate the key factors and structural relationships underlying the integration of Gerontechnology and spatial environments from the perspective of older adults. The effect strength in the INRM indicates the driving role of different dimensions within the system’s causal network, whereas the weights derived from DANP represent experts’ subjective evaluations of decision-making priorities, indicating which dimensions should be prioritized in policy or resource allocation. This study subsequently examines the causal relationships and relative priorities of each factor based on the effect strength ranking in the INRM.

#### 4.4.1 Gerontechnology application

Within the Gerontechnology Application dimension, Fire detection and Fall detection emerge as the most influential criteria, primarily functioning as early warning mechanisms and tools for preliminary incident assessment. Prior research has classified these technologies as essential for addressing adverse events (88). However, they may be insufficient to manage complex or multi-layered emergency scenarios independently.

Among the Gerontechnology Application criteria, Emergency notification dispatch ranks highest in priority and plays a pivotal role during emergency occurrences. The responsiveness of the emergency notification system directly influences the speed at which Automatic transmission of distress messages can relay information to frontline responders such as nurses, caregivers, or call center personnel. This responsiveness is a critical determinant of whether older adults can receive timely assistance in urgent situations. This finding aligns with the results of Melander-Wikman et al. (45), in which older participants expressed fear regarding the inability to obtain timely assistance and conveyed a strong preference for transmitting emergency alerts directly to professionals. Such preferences reflect concerns about unresponsive family members or misplaced external devices, risks that could delay help and ultimately undermine the core objective of Gerontechnology, to safeguard the safety and autonomy of older adults (88).

Beyond emergency response, Appointment and medication schedule reminders also serve a supportive role in facilitating long-term health monitoring within the Gerontechnology Application. Brignell et al. (89) demonstrated that such functions, when integrated as telehealth tools, enhance the effectiveness and efficiency of geriatric care. In particular, within the context of chronic disease management, these systems contribute to improved treatment outcomes and higher levels of patient satisfaction by supporting medication adherence and continuous health tracking.

#### 4.4.2 Age-friendly design

Although the Age-Friendly Design dimension includes only a single criterion (Fault-tolerant spatial configuration), its influence and strategic significance remain substantial. This criterion ranks first in overall priority within the system, thereby constituting a central component of the integrated framework. Fault-tolerant spatial configuration corresponds to the fifth principle of Universal Design, which emphasizes minimizing the risks and negative consequences associated with unintentional or erroneous user actions (90).

These findings underscore for decision-makers that while it is essential to adopt an age-friendly perspective in the integration of technology and spatial design, older adults should not be pathologized or treated as passive recipients of care (91, 92). Older individuals are frequently subjected to stigmatizing stereotypes such as associations with illness, dependency, or social irrelevance, which contribute to internalized shame and significantly hinder their acceptance of Gerontechnology based solutions (93–95). To address this challenge, applying fault-tolerant spatial configuration, a core principle of Universal Design enables the creation of environments that are inherently usable without the need for individual adaptation. This promotes stigma-free accessibility and enhances older adults’ willingness to engage with Gerontechnology enhanced spaces (90).

Specifically, fault-tolerant design can be implemented using redundancy and multimodal sensing, whereby motion detectors, wearable devices, and voice recognition systems can compensate for one another in the event of a malfunction (96). A similar approach has also been applied in fall detection systems, where multi-level confirmation mechanisms help to minimize false alarms (97). By embedding such redundancy and layered safeguards, age-friendly spaces can integrate smart technologies while ensuring resilience and user confidence (16).

#### 4.4.3 Public space

Within the Public Space dimension, Pavement material, width and level, Barrier-free access to and within buildings, and Separation of bicycles from pavement function as key Cause criteria, exerting strong influence within the system. These criteria play a critical role in ensuring that older adults can maintain unimpeded and safe mobility by minimizing the risks posed by physical barriers or suboptimal pavement conditions. The findings highlight the necessity for Public Space design to prioritize infrastructure elements that enhance usability and safety. As noted by Van Hoof et al. (98), pavement material, width, and level are fundamental components influencing not only the physical mobility of older adults but also their perceived safety. Wide and even sidewalks can significantly reduce the risk of falls, whereas uneven or narrow surfaces may become substantial barriers to outdoor movement among the older adults (99). The safety of sidewalks and bicycle lanes significantly impacts the spatial flow within open areas, thereby influencing older adults’ ease of movement and overall mobility (100). In particular, Ramírez-Saiz et al. (101) emphasized that the design of shared Public Spaces must account for anticipated user behaviors and safety risks associated with differences in movement speed. Moreover, a growing body of research suggests that the level of physical accessibility within Public Spaces, especially in terms of mobility, directly affects older adults’ comfort and ease in social engagement, which in turn shapes their willingness to interact with others in these environments (100, 102, 103).

These findings are consistent with those of Li et al. (104) and Yue et al. (105), which highlight the mediating roles of physical activity and social interaction in the relationship between the built environment and older adults’ health outcomes. Notably, although Outdoor safety enhancement measures and Pavement maintenance and dedication are positioned as Effect criteria within the Public Space dimension, their relatively high weights indicate that they rank among the most significant criteria, serving as core elements in ensuring age-friendly spatial design. This supports Turel et al.’s (106) assertion that the success of Public Spaces is not solely contingent upon their physical design and functionality, but also on their sustained vitality, which necessitates careful and ongoing maintenance. Therefore, policymakers should prioritize the long-term quality and safety management of infrastructure related to older adults’ mobility, thereby fostering greater willingness to engage in outdoor travel and supporting healthy aging.

#### 4.4.4 Living space

Within the Living Space dimension, Indoor natural light and Orientation and lighting are identified as critical criteria, exerting substantial influence on older adults’ comfort and mobility. As individuals age, vision deterioration becomes a progressive issue, with extended adaptation times to sudden light changes and heightened sensitivity to glare (107). Insufficient illumination and color temperature may restrict daily activities and hinder social participation among older adults. Furthermore, Sinoo et al. (108) highlighted that inadequate lighting in corridors, coupled with excessive contrast in brightness levels between corridors and Public Spaces, significantly increases the risk of falls. As such, the design and quality of lighting play a critical role in shaping the living environment, particularly in terms of enhancing safety, mobility, and well-being for older adults.

In contrast, the Roll-in shower is identified as having the lowest influence and priority, suggesting that while its presence is essential, decision-makers can improve and maintain its quality through other design criteria. This aligns with the findings of Aclan et al. (109) and Aplin et al. (110), who note that older adults are reluctant to adopt a “disability bathroom,” a concept often associated with negative stereotypes. Therefore, it is recommended that decision-makers incorporate personalized lighting and home decoration elements in the design of the Roll-in shower, creating an inclusive and non-stigmatized space that aligns with older adults’ preferences.

It is important to note that, despite being classified as an Effect criterion within the Living Space dimension, Comfortable ambient temperature ranks as the second most critical factor in the entire system. Liu et al. (111) highlighted that older adults are particularly sensitive to temperature changes, and extreme temperatures can severely impact both their comfort and health. Therefore, decision-makers should prioritize temperature regulation systems in Living Spaces, ensuring that indoor temperatures are consistently maintained within a comfortable range to prevent health issues caused by overheating or excessive cold, thereby supporting older adults’ functional independence and quality of life at home.

#### 4.4.5 Integration of results across dimensions

Combining the results across all dimensions, this study identifies Gerontechnology Application as the most influential dimension. However, other dimensions including Age-Friendly Design, Living Space, and Public Space are ranked with higher priority than Gerontechnology Application. The results indicate that in practical spatial planning, decision-makers may give priority to Age-Friendly Design (***D***_3_) to improve older adults’ everyday experiences, as it carries the highest weight and demonstrates strong policy feasibility. However, to ensure long term policy sustainability, it is essential to enhance the driving role of Gerontechnology Application (***D***_4_), as it plays a stronger causal role in the network and can indirectly facilitate improvements across other dimensions. At the same time, although the application of Gerontechnology can systematically enhance the utility of spatial integration, the Age-Friendly Design remains the most critical requirement in creating environments that truly support older adults’ well-being. This is consistent with recent studies addressing barriers to the implementation of Gerontechnology. For example, Han and Kim (29) emphasized that while modern technology continues to evolve, the integration of technological solutions with spatial environments must first and foremost address the daily needs of older adults, such as facilitating social interactions, mobility, and residential comfort. Without fulfilling these fundamental needs, the benefits of advanced technology for older adults remain limited. Additionally, the results reaffirm the position of Rafferty et al. (30), who argued that spatial constraints and uncertainties in user behavior are fundamental challenges to the successful integration of Gerontechnology in older adults care settings.

It should be noted that, although the Age-Friendly Design dimension includes only one factor after expert decision-making via FDM, it remains a critical factor within the causal structure and is regarded as a core Cause dimension. The results also help explain the adoption gap of Gerontechnology among older users. Pal et al. (112) observed that prior research on the integration of Gerontechnology into home environments often prioritized hedonic value as a key design factor. However, they found that “older adults do not perceive smart homes as a source of enjoyment.” This highlights a gap in current mainstream design priorities, which often fail to address the diverse health and technological needs of older adults, thereby resulting in lower adoption rates of Gerontechnology in this demographic (44). Therefore, Age-Friendly Design plays a pivotal role in the integration of technology and space, and decision-makers must give due consideration to this crucial aspect when developing age-friendly environments.

## 5 Conclusion

This study employed an integrated analysis of FDM and DANP to elucidate the causal structures and priority order among the dimensions, thereby developing an integrated framework. This framework addresses the high cost and maintenance challenges in the integration of Gerontechnology with spatial environments highlighted by Rafferty et al. (30). Moreover, the analysis revealed that Gerontechnology Application was the most influential dimension, whereas Age-Friendly Design emerged as the most critical dimension. These findings echo the observations of Peek et al. (16), who noted that the compatibility and adaptability of Gerontechnology with the environment are key factors influencing older adults’ willingness to adopt Gerontechnology. By combining causal and weight analyzes, this study offers a more cost effective and strategically oriented framework for the integration of Gerontechnology and spatial environments.

### 5.1 Theoretical implications

The theoretical contribution of this study lies in the development of a multi-criteria decision-making (MCDM) evaluation framework that integrates Gerontechnology with spatial environments and systematically elucidates the interdependencies and causal relationships among key factors. The findings not only broaden the scope of Gerontechnology but also fosters an interdisciplinary dialogue between environmental psychology, human factors engineering, and facilities management. For instance, the concept of Fault-tolerant spatial configuration in this study transcends the traditional focus on system stability in industrial products and is extended to encompass the spatial system’s ability to support older adults’ fundamental life functions and dignity. This theoretical implication, which translates technological resilience into caregiving potential, provides a fresh interpretative lens for understanding Gerontechnology and its integration with spatial environments.

Furthermore, through the application of FDM and DANP, this study extends the theoretical application of MCDM models to the field of aging space research. While MCDM has traditionally been used in industries such as engineering and manufacturing, this study introduces it to the domain of Gerontechnology and spatial design, demonstrating its capability to capture causal relationships and weightings between dimensions, thus providing a more explanatory and decision-supporting theoretical tool. By employing expert knowledge and experience for holistic judgments and system modeling, this study underscores the theoretical value and cost effectiveness of expert input during the exploratory phase of developing evaluation systems. This approach enables the systematic consideration of the evaluation system’s structure, grounded in experts’ professional knowledge and extensive practical experience, prior to the involvement of end users, thereby laying a foundation for subsequent empirical research. Moreover, this study incorporates multidimensional factors into the evaluation framework for Age-Friendly Design. This not only enriches the theoretical foundations of Gerontechnology Applications within spatial environments but also offers an operational model to inform the future development of human-centered smart space theories.

### 5.2 Managerial implications

This study employed collective expert judgments and multi-criteria decision-making methods to develop an evaluation framework for the application of Gerontechnology in spatial environments that is both operational and policy relevant. Particularly in contexts of limited resources or during the early stages of research, framework construction based on expert judgments can provide planning guidance and help avoid delays associated with large-scale field investigations. Furthermore, the results can serve as a reference for policymakers in promoting Gerontechnology initiatives and for the construction sector in implementing smart space standards, thereby fostering the sustainable development of age-friendly cities.

The findings indicate that designing age-friendly spaces cannot rely on single measures but instead requires simultaneously addressing real-time Gerontechnology Applications (e.g., fall detection, emergency alerts, medication reminders) and the structural safety of spatial configurations (e.g., fault-tolerant design of spaces, accessibility in public areas, indoor lighting and temperature control). This multidimensional approach can effectively mitigate risks for older adults in both daily living and emergency contexts, while enhancing their autonomy and sense of security.

Specifically, emergency detection and reporting systems (such as real-time notifications for falls and fires) represent not only technological advancements but also have a decisive impact on the safety and trust of older users. This necessitates that decision-makers integrate the automation and reliability of notification mechanisms into the early stages of spatial planning. The direct linkage to external professional support (e.g., medical units) should be considered a critical facility rather than an optional addition. In the Public Space dimension, pavement maintenance, pedestrian flow separation, and barrier-free facilities have been established as key management measures that enhance older adults’ willingness to engage in physical activity and social participation. Facility managers should exceed minimum standards and proactively assess safety risks along mobility paths and in high-traffic areas, regularly updating design configurations to address emerging needs. This same management logic applies to Living Spaces, particularly regarding indoor natural lighting and temperature and humidity regulation. Given that older adults have a lower sensitivity to these environmental factors, environmental control systems must be implemented for fine-tuning and predictive management, reducing discomfort and mitigating the risk of falls.

Additionally, this study highlights that the application of Gerontechnology spans multiple dimensions, including Living Spaces, Public Spaces, and Age-Friendly Design, with significant interdependencies and interactions between these dimensions and their respective criteria. Therefore, the integration of Gerontechnology with Age-Friendly spaces requires collaboration across various sectors. A key consideration for decision-makers across these fields is the necessity of adopting flexible design principles and incorporating fault-tolerant capabilities as the central approach for evaluation and integration, with universal design being one example. If decision-makers prioritize only technical configurations without considering the stability, flexibility, and adaptability of the technology and design, this could lead to a lack of compatibility between the technology and the physical environment, thus reducing the overall effectiveness of the space and diminishing older adults’ willingness to adopt the technology.

In other words, decision-makers should move beyond the traditional approach of one-time construction, adopting flexible and upgradeable strategic planning models. For instance, when integrating technologies such as perception devices, remote health monitoring, and fall detection systems into physical spaces, it is essential to consider not only ease of use and scalability but also future maintenance to minimize barriers for older adults. This will ultimately enhance their autonomy and increase the actual usage rates of these technologies. This ensures that the integration of Gerontechnologies and spatial planning can generate true synergies, enabling older adults to live independently for extended periods and supporting the development of a sustainable, human-centered, age-friendly environment.

### 5.3 Limitation and future research

Although this study integrates Gerontechnology and spatial environments through a hybrid MCDM model to construct a systematic analytical framework, several limitations remain. Although the model effectively reflects the overall trends and priorities of older adults’ spatial needs, regional variations may be considerable. The disparities between urban and rural populations are particularly pronounced, not only with respect to infrastructure accessibility but also in terms of attitudes toward technological interventions, perceptions, and lifestyle patterns. Therefore, future research should pursue two complementary directions.

First, the multidimensional nature of Age-Friendly Design can be further delineated into sub-dimensions such as autonomy, dignity, privacy, and social connectedness. Second, greater attention should be devoted to contextual diversity through region-specific and culturally sensitive analyzes. Such contextualized adjustments would enhance the framework’s responsiveness and practical applicability, ensuring that Gerontechnology design strategies are not only theoretically robust but also adaptable to the lived realities of older adults across diverse environments.

In addition, this study primarily relied on expert experience to establish the indicator system and causal structure. At the early research stage, this approach facilitated the rapid identification of key influencing factors and ensured theoretical as well as professional rigor, thus laying a solid foundation for subsequent fieldwork and participatory research. However, because older adults often face constraints such as time and energy, this study did not directly involve end users (i.e., older adults or caregivers) for empirical validation at this stage. Therefore, future research and practical applications should incorporate user experiences to more comprehensively reflect actual needs and usage contexts.

## Data Availability

The raw data supporting the conclusions of this article will be made available by the authors, without undue reservation.

## References

[1] Li M Woolrych R. Experiences of older people and social inclusion in relation to smart “age-friendly” cities: a case study of Chongqing, China. Front Public Health. (2021) 9:779913. doi: 10.3389/fpubh.2021.779913, PMID: 34988053 PMC8721664

[2] Cheng M An S Cheung CF Leung Z Chun TK. Gerontechnology acceptance by older adults and their satisfaction on its servitization in Hong Kong. Behav Inform Technol. (2023) 42:2932–51. doi: 10.1080/0144929X.2022.2151936

[3] Peine A Marshall B Martin W Neven L. Socio-gerontechnology: Interdisciplinary critical studies of ageing and technology. London, New York: Routledge (2021).

[4] Bouma H Fozard JL Bouwhuis DG Taipale V. Gerontechnology in perspective. Geron. (2007) 6:190–216. doi: 10.4017/gt.2007.06.04.003.00

[5] Halicka K Surel D. Gerontechnology—new opportunities in the service of older adults. Eng Manag Prod Serv. (2021) 13:114–26. doi: 10.2478/emj-2021-0025

[6] Fozard JL Rietsema J Bouma H Graafmans JAM. Gerontechnology: creating enabling environments for the challenges and opportunities of aging. Educ Gerontol. (2000) 26:331–44. doi: 10.1080/036012700407820

[7] Parra-Rodríguez L Reyes-Ramírez ED Pérez-Sanpablo AI. Gerontechnology. In: Aging research—Methodological issues. Cham: Springer Nature Switzerland (2024). 197–210.

[8] Pilotto A Volta E Barbagelata M Custodero C. Gerontechnology: definitions and classification In: Pilotto A Volta E Barbagelata M Custodero C, editors. Gerontechnology. A clinical perspective. Cham: Springer International Publishing (2023). 3–14.

[9] Ofli F Kurillo G Obdržálek Š Bajcsy R Jimison HB Pavel M. Design and evaluation of an interactive exercise coaching system for older adults: lessons learned. IEEE J Biomed Health Inform. (2015) 20:201–12. doi: 10.1109/JBHI.2015.2391671 25594988 PMC4835340

[10] Peine A Rollwagen I Neven L. The rise of the “innosumer”—rethinking older technology users. Technol Forecast Soc Change. (2014) 82:199–214. doi: 10.1016/j.techfore.2013.06.013

[11] Guner H Acarturk C. The use and acceptance of ICT by senior citizens: a comparison of technology acceptance model (TAM) for elderly and young adults. Univer Access Inf Soc. (2020) 19:311–30. doi: 10.1007/s10209-018-0642-4

[12] Wang S Bolling K Mao W Reichstadt J Jeste D Kim HC. Technology to support aging in place: older adults’ perspectives. Healthcare. (2019) 7:60. doi: 10.3390/healthcare7020060, PMID: 30974780 PMC6627975

[13] Chen K Chan AHS. Gerontechnology acceptance by elderly Hong Kong Chinese: a senior technology acceptance model (STAM). Ergonomics. (2014) 57:635–52. doi: 10.1080/00140139.2014.895855, PMID: 24655221

[14] Lee C Coughlin JF. Perspective: older adults' adoption of technology: an integrated approach to identifying determinants and barriers. J Prod Innov Manag. (2015) 32:747–59. doi: 10.1111/jpim.12176

[15] Macedo IM. Predicting the acceptance and use of information and communication technology by older adults: an empirical examination of the revised UTAUT2. Comput Human Behav. (2017) 75:935–48. doi: 10.1016/j.chb.2017.06.013

[16] Peek ST Luijkx KG Rijnaard MD Nieboer ME Van Der Voort CS Aarts S. Older adults' reasons for using technology while aging in place. Gerontology. (2016) 62:226–37. doi: 10.1159/000430949, PMID: 26044243

[17] Carrera L. Active aging and urban policies: the space as an instrument for an inclusive and sustainable city. Front Sociol. (2023) 8:1257926. doi: 10.3389/fsoc.2023.1257926, PMID: 38146316 PMC10749359

[18] Peine A Neven L. The co-constitution of ageing and technology–a model and agenda. Ageing Soc. (2021) 41:2845–66. doi: 10.1017/S0144686X20000641

[19] Jiravanichkul S Pinich S Sreshthaputra A Jarutach T. The development of a well-being environment and age-friendly communities assessment criteria using the analytic hierarchy process: a case of Thailand. Nakhara J Environ Des Plann. (2024) 23:416–6. doi: 10.54028/NJ202423416

[20] Zarghami E Sharghi A Olfat M Salehi Kousalari F. Using multi-criteria decision-making method (MCDM) to study quality of life variables in the design of senior residences in Iran. Ageing Int. (2018) 43:279–96. doi: 10.1007/s12126-017-9308-4

[21] Weck M Humala I Tamminen P Ferreira FA. Supporting sustainable development using multiple criteria decision aid: towards an age-friendly smart living environment. In: Multiple criteria decision making for sustainable development: Pursuing economic growth, environmental protection and social cohesion. Cham: Springer International Publishing (2022). 151–73.

[22] Ma C Guerra-Santin O Mohammadi M. Smart home modification design strategies for ageing in place: a systematic review. J Housing Built Environ. (2022) 37:625–51. doi: 10.1007/s10901-021-09888-z

[23] Thomése F Broese van Groenou M. Adaptive strategies after health decline in later life: increasing the person-environment fit by adjusting the social and physical environment. Eur J Ageing. (2006) 3:169–77. doi: 10.1007/s10433-006-0038-9, PMID: 28794761 PMC5546375

[24] Trecartin SM Cummings SM. Systematic review of the physical home environment and the relationship to psychological well-being among community-dwelling older adults. J Gerontol Soc Work. (2018) 61:567–82. doi: 10.1080/01634372.2018.1463339, PMID: 29668403

[25] Anderberg P. Gerontechnology, digitalization, and the silver economy. XRDS. (2020) 26:46–9. doi: 10.1145/3383388

[26] Kohlbacher F Herstatt C Levsen N. Golden opportunities for silver innovation: how demographic changes give rise to entrepreneurial opportunities to meet the needs of older people. Technovation. (2015) 39-40:73–82. doi: 10.1016/j.technovation.2014.05.002, PMID: 40972618

[27] Stamate A Marzan MD Velciu M Paul C Spiru L. Advancing user-centric design and technology adoption for aging populations: a multifaceted approach. Front Public Health. (2024) 12:1469815. doi: 10.3389/fpubh.2024.1469815, PMID: 39712308 PMC11659139

[28] Wilson C Hargreaves T Hauxwell-Baldwin R. Smart homes and their users: a systematic analysis and key challenges. Pers Ubiquit Comput. (2015) 19:463–76. doi: 10.1007/s00779-014-0813-0

[29] Han MJN Kim MJ. A critical review of the smart city in relation to citizen adoption towards sustainable smart living. Habitat Int. (2021) 108:102312. doi: 10.1016/j.habitatint.2021.102312

[30] Rafferty J Nugent CD Liu J Chen L. From activity recognition to intention recognition for assisted living within smart homes. IEEE Trans Hum Mach Syst. (2017) 47:368–79. doi: 10.1109/THMS.2016.2641388

[31] Han MJN Kim MJ. A smart social sustainability model for smart city to enhance human context experience. Cities. (2025) 159:105788. doi: 10.1016/j.cities.2025.105788

[32] Chen FH Hsu TS Tzeng GH. A balanced scorecard approach to establish a performance evaluation and relationship model for hot spring hotels based on a hybrid MCDM model combining DEMATEL and ANP. Int J Hosp Manag. (2011) 30:908–32. doi: 10.1016/j.ijhm.2011.02.001

[33] Saaty T. L. (1996). Decision making with dependence and feedback: The analytic network process (4922). Pittsburgh: RWS publications.

[34] Lee AT Ramasamy RK Subbarao A. Understanding psychosocial barriers to healthcare technology adoption: a review of TAM technology acceptance model and unified theory of acceptance and use of technology and UTAUT frameworks. Healthcare. (2025) 13:250. doi: 10.3390/healthcare13030250 39942440 PMC11816427

[35] Shin HR Um SR Yoon HJ Choi EY Shin WC Lee HY. Comprehensive senior technology acceptance model of daily living assistive technology for older adults with frailty: cross-sectional study. J Med Internet Res. (2023) 25:e41935. doi: 10.2196/41935, PMID: 37036760 PMC10131916

[36] Yang CC Li CL Yeh TF Chang YC. Assessing older adults’ intentions to use a smartphone: using the Meta–unified theory of the acceptance and use of technology. Int J Environ Res Public Health. (2022) 19:5403. doi: 10.3390/ijerph19095403, PMID: 35564798 PMC9102817

[37] Yang HJ Lee JH Lee W. Factors influencing health care technology acceptance in older adults based on the technology acceptance model and the unified theory of acceptance and use of technology: meta-analysis. J Med Internet Res. (2025) 27:e65269. doi: 10.2196/65269, PMID: 40153796 PMC11992498

[38] Özsungur F. A research on the effects of successful aging on the acceptance and use of technology of the elderly. Assist Technol. (2022) 34:77–90. doi: 10.1080/10400435.2019.1691085, PMID: 31710261

[39] Huang L Li X Li X Wen Y Yuan F. Research on the influencing factors of Gerontechnology acceptance by seniors: a case study of Beijing elderly citizens. Innov Dev Policy. (2021) 3:91–109.

[40] Joo S Kim SH Lee C Kim CO Lim YM Jun HJ. Who matters for the subjective perceptions toward gerontechnology? Innov Aging. (2021) 5:661–1. doi: 10.1093/geroni/igab046.2497

[41] Murciano-Hueso A Martín-Lucas J Serrate González S Torrijos Fincias P. Use and perception of gerontechnology: differences in a group of Spanish older adults. Qual Ageing Older Adults. (2022) 23:114–28. doi: 10.1108/QAOA-02-2022-0010

[42] Huang SW Liou JJ Cheng SH Tang W Ma JC Tzeng GH. The key success factors for attracting foreign investment in the post-epidemic era. Axioms. (2021) 10:140. doi: 10.3390/axioms10030140

[43] Teh PL Wang H Phang CW Chan AH Butt HP. Important but not for me: understanding older adults’ resistance to gerontechnology In: Teh PL Wang H Phang CW Chan AH Butt HP, editors. Emerging technologies in business: Innovation strategies for competitive advantage. Singapore: Springer Nature Singapore (2024). 11–45.

[44] Huang G Oteng SA. Gerontechnology for better elderly care and life quality: a systematic literature review. Eur J Ageing. (2023) 20:27. doi: 10.1007/s10433-023-00776-9, PMID: 37347277 PMC10287881

[45] Melander-Wikman A Fältholm Y Gard G. Safety vs. privacy: elderly persons’ experiences of a mobile safety alarm. Health Soc Care Community. (2008) 16:337–46. doi: 10.1111/j.1365-2524.2007.00743.x, PMID: 18613909

[46] Thrift N. On the determination of social action in space and time. Environment and planning D: Society and space 1.1. (1983) 23–57. doi: 10.1068/d010023

[47] Fozard J. L. Graafmans J. A. Rietsema J. Bouma H. van Berlo G. M. W. (1993). Aging and ergonomics: The challenges of individual differences and environmental change. The Netherlands: University of Groningen, Traffic Research Centre, Haren.

[48] World Health Organization. Global age-friendly cities: a guide. Geneva, Switzerland: World Health Organization. (2007).

[49] Yen IH Anderson LA. Built environment and mobility of older adults: important policy and practice efforts. J Am Geriatr Soc. (2012) 60:951–6. doi: 10.1111/j.1532-5415.2012.03949.x, PMID: 22568533 PMC4534005

[50] Age Friendly Ireland. (2021). Age friendly homes rating checklist. Age friendly homes rating tool June 2021. Available online at: https://agefriendlyireland.ie/wp-content/uploads/2021/06/AFI-Homes-Rating-Checklist.pdf (Accessed July 10, 2025).

[51] World Health Organization (2018) WHO housing and health guidelines. Geneva, Switzerland: World Health Organization.

[52] Grazuleviciute-Vileniske I Seduikyte L Teixeira-Gomes A Mendes A Borodinecs A Buzinskaite D. Aging, living environment, and sustainability: what should be taken into account? Sustainability. (2020) 12:1853. doi: 10.3390/su12051853

[53] Gurung S Chaudhury H. Relationship-centered care for older adults in long-term care homes: a scoping review. J Appl Gerontol. (2025) 44:1513–32. doi: 10.1177/07334648241309761, PMID: 39787049 PMC12335636

[54] Morhardt D Spira M. From person-centered care to relational centered care. Generations. (2013) 37:37–44.

[55] Rutten JE Backhaus R Verbeek H de Vries E Hamers JP Sion KY. Improving relationship-centered care through evaluation meetings with the resident-family-caregiver triad in nursing homes: a qualitative study. BMC Health Serv Res. (2025) 25:296. doi: 10.1186/s12913-025-12425-1 39987061 PMC11846192

[56] Young HM. Relationship-Centered care: a path to improving nursing home outcomes. Res Gerontol Nurs. (2023) 16:2–3. doi: 10.3928/19404921-20230105-01, PMID: 36692434

[57] Brownie S Nancarrow S. Effects of person-centered care on residents and staff in aged-care facilities: a systematic review. Clin Interv Aging. (2013) 8:1–10. doi: 10.2147/CIA.S38589, PMID: 23319855 PMC3540911

[58] Morgan S Yoder LH. A concept analysis of person-centered care. J Holist Nurs. (2012) 30:6–15. doi: 10.1177/0898010111412189, PMID: 21772048

[59] Boot W Charness N Czaja SJ Rogers WA. Designing for older adults: Case studies, methods, and tools. Boca Raton: CRC Press (2020).

[60] Chang D Gu Z Li F Jiang R. A user-centric smart product-service system development approach: a case study on medication management for the elderly. Adv Eng Inform. (2019) 42:100979. doi: 10.1016/j.aei.2019.100979

[61] Mao Q Teh PL Wang SJ Wang H. Freedom to personalize walking aids: a user-centric design framework for age-friendly smart canes. Int J Hum Comput Interact. (2025) 1–16. doi: 10.1080/10447318.2025.2526577

[62] Alqahtani S Joseph J Dicianno B Layton NA Toro ML Ferretti E. Stakeholder perspectives on research and development priorities for mobility assistive-technology: a literature review. Disabil Rehabil Assist Technol. (2021) 16:362–76. doi: 10.1080/17483107.2019.1650300, PMID: 31535934

[63] Satpathy L. (2006). Smart housing: Technology to aid aging in place-new opportunities and challenges. M.S. Thesis, Starkville, Mississippi State University.

[64] Almusaed A Yitmen I Almssad A. Enhancing smart home design with AI models: a case study of living spaces implementation review. Energies. (2023) 16:2636. doi: 10.3390/en16062636

[65] Tivatansakul S. Tanupaprungsun S. Areekijseree K. Achalakul T. Hirasawa K. Sawada S., … & Ohkura M. (2012). The intelligent space for the elderly—implementation of fall detection algorithm. In 2012 Proceedings of SICE Annual Conference (SICE) (pp. 1944–1949). Akita: IEEE.

[66] Ge R. Shan Z. Kou H. (2011). An intelligent surveillance system based on motion detection. In 2011 4th IEEE International Conference on Broadband Network and Multimedia Technology (306–309). Shenzhen: IEEE.

[67] Jeng TB. Fuzzy assessment model for maturity of software organization in improving its staff’s capability (doctoral dissertation). Taipei: National Taiwan University of Science and Technology (2001).

[68] Renthlei E George A. Toward holistic neighbourhood sustainability assessment: integrating fuzzy Delphi method for sustainable indicator selection for Aizawl city. Loc Environ. (2025) 30:622–41. doi: 10.1080/13549839.2024.2413080

[69] Tsai HC Lee AS Lee HN Chen CN Liu YC. An application of the fuzzy Delphi method and fuzzy AHP on the discussion of training indicators for the regional competition, Taiwan national skills competition, in the trade of joinery. Sustainability. (2020) 12:4290. doi: 10.3390/su12104290

[70] Wang Y Yeo GT. Intermodal route selection for cargo transportation from Korea to Central Asia by adopting fuzzy Delphi and fuzzy ELECTRE I methods. Marit Policy Manag. (2018) 45:3–18. doi: 10.1080/03088839.2017.1319581

[71] Zheng XY Zhu BW Wang K Tzeng GH Xiong L. Decoding authenticity judgments in ethnic restaurants: a hybrid approach for bridging cultural gatekeeper and adventurer perspectives. Int J Hosp Manag. (2026) 132:104390. doi: 10.1016/j.ijhm.2025.104390

[72] Gabus A. Fontela E. (1973) Perceptions of the world problematique: Communication procedure, communicating with those bearing collective responsibility. Geneva, Switzerland: Battelle Geneva Research Centre.

[73] Gabus A Fontela E. The DEMATEL observer. Battelle Geneva Research Center, Geneva, Switzerland (1976).

[74] Hsu CH Wang FK Tzeng GH. The best vendor selection for conducting the recycled material based on a hybrid MCDM model combining DANP with VIKOR. Resour Conserv Recycl. (2012) 66:95–111. doi: 10.1016/j.resconrec.2012.02.009

[75] Qu GB Zhao TY Zhu BW Tzeng GH Huang SL. Use of a modified DANP-mV model to improve quality of life in rural residents: the empirical case of Xingshisi village, China. Int J Environ Res Public Health. (2019) 16:153. doi: 10.3390/ijerph16010153, PMID: 30626073 PMC6339206

[76] Shen KY Yan MR Tzeng GH. Combining VIKOR-DANP model for glamor stock selection and stock performance improvement. Knowl-Based Syst. (2014) 58:86–97. doi: 10.1016/j.knosys.2013.07.023

[77] Wang K Li XY Zhu BW Xiong L Tzeng GH. A data mining approach to explore the causal rules between environmental conditions of neighborhood parks and seniors' satisfaction. Cities. (2025) 162:105897. doi: 10.1016/j.cities.2025.105897

[78] Zhu BW Xiao YH Zheng WQ Xiong L He XY Zheng JY. A hybrid multiple-attribute decision-making model for evaluating the esthetic expression of environmental design schemes. SAGE Open. (2022) 12:21582440221087268. doi: 10.1177/21582440221087268

[79] Zhu BW Zhang JR Tzeng GH Huang SL Xiong L. Public open space development for elderly people by using the DANP-V model to establish continuous improvement strategies towards a sustainable and healthy aging society. Sustainability. (2017) 9:420. doi: 10.3390/su9030420

[80] Saaty TL. Decision making—the analytic hierarchy and network processes (AHP/ANP). J Syst Sci Syst Eng. (2004) 13:1–35. doi: 10.1007/s11518-006-0151-5

[81] Li R Lu Y. Toward a resilient and smart city: analysis on enablers for smart city resilience using an integrated DEMATEL–ISM–ANP method. Technol Forecast Soc Change. (2025) 215:124081. doi: 10.1016/j.techfore.2025.124081

[82] Shao QG Jiang CC Lo HW Liou JJ. Establishing a sustainable development assessment framework for a smart city using a hybrid Z-fuzzy-based decision-making approach. Clean Techn Environ Policy. (2023) 25:3027–44. doi: 10.1007/s10098-023-02547-7

[83] Liu Y Li M Chen Y Tzeng GH. Evaluation of and improvement planning for smart homes using rough knowledge-based rules on a hybrid multiple attribute decision-making model. Soft Comput. (2020) 24:7781–800. doi: 10.1007/s00500-019-04396-3

[84] Li SJ Luo YF Liu ZC Xiong L Zhu BW. Exploring strategies for improving green open spaces in old downtown residential communities from the perspective of public health to enhance the health and well-being of the aged. J Healthc Eng. (2021) 2021:1–20. doi: 10.1155/2021/5547749, PMID: 35126893 PMC8814349

[85] Feng IM Chen JH Zhu BW Xiong L. Assessment of and improvement strategies for the housing of healthy elderly: improving quality of life. Sustainability. (2018) 10:722. doi: 10.3390/su10030722

[86] Lin PJ Shiue YC Tzeng GH Huang SL. Developing a sustainable long-term ageing health care system using the DANP-mV model: empirical case of Taiwan. Int J Environ Res Public Health. (2019) 16:1349. doi: 10.3390/ijerph16081349, PMID: 30991706 PMC6518165

[87] Zheng WQ Cheung SM Zhu BW Xiong L Tzeng GH. A hybrid multi-attribute decision-making model for the systematic evaluation of exoticism-themed retail spaces from the perspective of consumer experience. J Retail Consum Serv. (2024) 79:103848. doi: 10.1016/j.jretconser.2024.103848

[88] Ludwig W Wolf KH Duwenkamp C Gusew N Hellrung N Marschollek M. Health-enabling technologies for the elderly–an overview of services based on a literature review. Comput Methods Prog Biomed. (2012) 106:70–8. doi: 10.1016/j.cmpb.2011.11.001, PMID: 22115611

[89] Brignell M Wootton R Gray L. The application of telemedicine to geriatric medicine. Age Ageing. (2007) 36:369–74. doi: 10.1093/ageing/afm045, PMID: 17449535

[90] Carr K Weir PL Azar D Azar NR. Universal design: a step toward successful aging. J Aging Res. (2013) 2013:324624. doi: 10.1155/2013/324624, PMID: 23431446 PMC3570931

[91] McGinley C Myerson J Briscoe G Carroll S. Towards an age-friendly design lens. J Popul Ageing. (2022) 15:541–56. doi: 10.1007/s12062-022-09367-5

[92] Story MF. Maximizing usability: the principles of universal design. Assist Technol. (1998) 10:4–12. doi: 10.1080/10400435.1998.10131955, PMID: 10181150

[93] Li C Lee CF Xu S. Stigma threat in design for older adults: exploring design factors that induce stigma perception. Int J Des. (2020) 14:51–64.

[94] Todorović T. Millennials and elderly people: discrimination or fundamental misunderstanding?. In: Elderly people and discrimination: Prevention and reaction. Institute of Criminological and Sociological ResearchVojvodina Bar Association, Vladimir Beljanski. (2023). 589.

[95] Wei Y Chen J. Sustainable Design for the Silver Society: developing the silver model for Gerontechnology product innovation. Sustainability. (2025) 17:42. doi: 10.3390/su17010042, PMID: 40881048

[96] Rashidi P Mihailidis A. A survey on ambient-assisted living tools for older adults. IEEE J Biomed Health Inform. (2013) 17:579–90. doi: 10.1109/JBHI.2012.2234129, PMID: 24592460

[97] Igual R Medrano C Plaza I. Challenges, issues and trends in fall detection systems. Biomed Eng Online. (2013) 12:66. doi: 10.1186/1475-925X-12-66, PMID: 23829390 PMC3711927

[98] Van Hoof J Dikken J van Staalduinen WH van der Pas S van den Hoven RF Hulsebosch-Janssen LM. Towards a better understanding of the sense of safety and security of community-dwelling older adults. The case of the age-friendly city of the Hague. Int J Environ Res Public Health. (2022) 19:3960. doi: 10.3390/ijerph19073960 35409643 PMC8997810

[99] Younes SR Marques B McIntosh J. Public spaces for older people: a review of the relationship between public space and quality of life. Sustainability. (2024) 16:4583. doi: 10.3390/su16114583

[100] Yung EH Conejos S Chan EH. Public open spaces planning for the elderly: the case of dense urban renewal districts in Hong Kong. Land Use Policy. (2016) 59:1–11. doi: 10.1016/j.landusepol.2016.08.022

[101] Ramírez-Saiz A Baquero Larriva MT Jiménez Martín D Alonso A. Enhancing urban mobility for all: the role of universal design in supporting social inclusion for older adults and people with disabilities. Urban Sci. (2025) 9:46. doi: 10.3390/urbansci9020046

[102] Fadda G Cortés A Olivi A Tovar M. The perception of the values of urban space by senior citizens of Valparaiso. J Aging Stud. (2010) 24:344–57. doi: 10.1016/j.jaging.2010.07.001

[103] Kwok JYC Ng KCH. User friendly living environmental research and Design for Older People. In: Designing inclusive futures. London: Springer London (2008). 261–72.

[104] Li J Tian L Ouyang W. Exploring the relationship between neighborhood-built environment and elderly health: a research based on heterogeneity of age and gender groups in Beijing. Front Public Health. (2022) 10:882361. doi: 10.3389/fpubh.2022.882361, PMID: 35712265 PMC9194851

[105] Yue Y Yang D Owen N Van Dyck D. The built environment and mental health among older adults in Dalian: the mediating role of perceived environmental attributes. Soc Sci Med. (2022) 311:115333. doi: 10.1016/j.socscimed.2022.115333, PMID: 36084518

[106] Turel HS Yigit EM Altug I. Evaluation of elderly people's requirements in public open spaces: a case study in Bornova District (Izmir, Turkey). Build Environ. (2007) 42:2035–45. doi: 10.1016/j.buildenv.2006.03.004

[107] Che L Zhang J Liu J Ma M Si X An J. Interior luminous environment for the elderly: recommended values of lighting parameters and comfort discriminant model. Build Environ. (2025) 274:112787. doi: 10.1016/j.buildenv.2025.112787

[108] Sinoo MM Van Hoof J Kort HS. Light conditions for older adults in the nursing home: assessment of environmental illuminances and colour temperature. Build Environ. (2011) 46:1917–27. doi: 10.1016/j.buildenv.2011.03.013

[109] Aclan R George S Laver K. Common home hazards among healthy older aged adults and potential modifications required for age-friendly housing. Aust Occup Ther J. (2024) 71:213–25. doi: 10.1111/1440-1630.12918, PMID: 38016761

[110] Aplin T De Jonge D Gustafsson L. Understanding the dimensions of home that impact on home modification decision making. Aust Occup Ther J. (2013) 60:101–9. doi: 10.1111/1440-1630.12022, PMID: 23551003

[111] Liu Y Li X Zeng Y. Thermal comfort and its impact on the quality of life for elderly residents: a review. Build Environ. (2016) 105:120–8. doi: 10.1016/j.buildenv.2016.06.001

[112] Pal D Funilkul S Vanijja V Papasratorn B. Analyzing the elderly users’ adoption of smart-home services. IEEE Access. (2018) 6:51238–52. doi: 10.1109/ACCESS.2018.2869599

[113] Lin CC Chiu MJ Hsiao CC Lee RG Tsai YS. Wireless health care service system for elderly with dementia. IEEE Trans Inf Technol Biomed. (2006) 10:696–704. doi: 10.1109/TITB.2006.874196, PMID: 17044403

[114] Boulos MNK Rocha A Martins A Vicente ME Bolz A Feld R. CAALYX: a new generation of location-based services in healthcare. Int J Health Geogr. (2007) 6:9. doi: 10.1186/1476-072X-6-9, PMID: 17352802 PMC1828720

[115] Alwan M Dalal S Mack D Kell S Turner B Leachtenauer J. Impact of monitoring technology in assisted living: outcome pilot. IEEE Trans Inf Technol Biomed. (2006) 10:192–8. doi: 10.1109/TITB.2005.855552, PMID: 16445264

[116] Essén A Conrick M. New e-service development in the homecare sector: beyond implementing a radical technology. Int J Med Inform. (2008) 77:679–88. doi: 10.1016/j.ijmedinf.2008.02.001, PMID: 18514021

[117] Kleinpell RM Avitall B. Integrating telehealth as a strategy for patient management after discharge for cardiac surgery: results of a pilot study. J Cardiovasc Nurs. (2007) 22:38–42. doi: 10.1097/00005082-200701000-00006, PMID: 17224696

[118] Van Hoof J Kort HSM Rutten PGS Duijnstee MSH. Ageing-in-place with the use of ambient intelligence technology: Perspectives of older users. International journal of medical informatics. (2011) 80:310–31. 21439898 10.1016/j.ijmedinf.2011.02.010

[119] Jacelon CS Hanson A. Older adults’ participation in the development of smart environments: An integrated review of the literature. Geriatric Nursing, 34:116–21. 10.1016/j.gerinurse.2012.11.001 23276642

[120] Zagler WL Paul P Marjo R. Ambient assisted living systems-the conflicts between technology, acceptance, ethics and privacy. Schloss Dagstuhl–Leibniz-Zentrum für Informatik, (2008).

[121] Afacan Y Cigdem E. An interdisciplinary heuristic evaluation method for universal building design. Applied Ergonomics. (2009) 40:731–44. 18775531 10.1016/j.apergo.2008.07.002

[122] Mustaquim MM. A study of universal design in everyday life of elderly adults. Procedia Computer Science. (2015) 67:57–66.

