# Supplementary material for: Developing age-friendly spaces through a gerontechnological lens: a systemic framework based on FDM-DANP analysis
Source: Front Med (Lausanne). 2025 Oct 13;12:1681486. doi: 10.3389/fmed.2025.1681486 (PMC12554651; doi:10.3389/fmed.2025.1681486)
Supplement: Supplementary file 1 [file Data_Sheet_1.docx]

**Appendix**

**Appendix 1. FDM Questionnaire**

**Survey on the Key Factors of Gerontechnology and Spatial Integration**

Dear Participant,

This questionnaire focuses on identifying and evaluating the key factors of gerontechnology and spatial integration. The purpose of this study is to collect expert opinions through a structured survey in order to determine critical factors that influence planning and management practices. The results are expected to prevent resource waste caused by unplanned management and to provide valuable decision-making support for relevant agencies in planning and design. This will help ensure more efficient and evidence-based resource allocation for the development and subsequent management of intelligent spaces in suburban areas. Your feedback is highly valuable and will greatly enhance the quality and reliability of this research. Please be assured that the information you provide will be used exclusively for academic analysis and will be kept strictly confidential.

1. **Descriptions of Potential Dimensions and Associated Criteria**

(As shown in Table 1)

1. **Assessment of Potential Key Factors**

| **No.** | **Please rate each item on a scale from: 1 (very important) to 5 (not important at all)** | **Level** |
| --- | --- | --- |
| *I*_1_ | I think that **Cleanliness of public spaces** for Gerontechnology and Spatial Integration, is |  |
| *I*_2_ | I think that **Quantity and safety of green spaces and outdoor seating** for Gerontechnology and Spatial Integration, is |  |
| *I*_3_ | I think that **Sidewalk maintenance and exclusivity** for Gerontechnology and Spatial Integration, is |  |
| *I*_4_ | I think that **Sidewalk material, width, and leveling** for Gerontechnology and Spatial Integration, is |  |
| *I*_5_ | I think that **Number and safety of pedestrian crossings** for Gerontechnology and Spatial Integration, is |  |
| *I*_6_ | I think that **Driver yielding behavior** for Gerontechnology and Spatial Integration, is |  |
| *I*_7_ | I think that **Separation of bicycles from pedestrian paths** for Gerontechnology and Spatial Integration, is |  |
| *I*_8_ | I think that **Outdoor safety enhancement measures** for Gerontechnology and Spatial Integration, is |  |
| *I*_9_ | I think that **Service concentration** for Gerontechnology and Spatial Integration, is |  |
| *I*_10_ | I think that **Special customer service arrangements** for Gerontechnology and Spatial Integration, is |  |
| *I*_11_ | I think that **Indoor and outdoor signage** for Gerontechnology and Spatial Integration, is |  |
| *I*_12_ | I think that **Indoor and outdoor seating** for Gerontechnology and Spatial Integration, is |  |
| *I*_13_ | I think that **Indoor and outdoor restrooms** for Gerontechnology and Spatial Integration, is |  |
| *I*_14_ | I think that **Indoor and outdoor accessible features** for Gerontechnology and Spatial Integration, is |  |
| *I*_15_ | I think that **Guest room** for Gerontechnology and Spatial Integration, is |  |
| *I*_16_ | I think that **Building energy rating** for Gerontechnology and Spatial Integration, is |  |
| *I*_17_ | I think that **Attic insulation** for Gerontechnology and Spatial Integration, is |  |
| *I*_18_ | I think that **Thermal water tanks** for Gerontechnology and Spatial Integration, is |  |
| *I*_19_ | I think that **Wall insulation** for Gerontechnology and Spatial Integration, is |  |
| *I*_20_ | I think that **Low-maintenance heating systems** for Gerontechnology and Spatial Integration, is |  |
| *I*_21_ | I think that **Outdoor storage space** for Gerontechnology and Spatial Integration, is |  |
| *I*_22_ | I think that **Heating expenditure ratio** for Gerontechnology and Spatial Integration, is |  |
| *I*_23_ | I think that **Renewable energy sources** for Gerontechnology and Spatial Integration, is |  |
| *I*_24_ | I think that **House orientation and daylighting** for Gerontechnology and Spatial Integration, is |  |
| *I*_25_ | I think that **Accessible heating controls** for Gerontechnology and Spatial Integration, is |  |
| *I*_26_ | I think that **Roll-in shower accessibility** for Gerontechnology and Spatial Integration, is |  |
| *I*_27_ | I think that **Bathroom proximity** for Gerontechnology and Spatial Integration, is |  |
| *I*_28_ | I think that **Indoor natural light** for Gerontechnology and Spatial Integration, is |  |
| *I*_29_ | I think that **Comfortable ambient temperature** for Gerontechnology and Spatial Integration, is |  |
| *I*_30_ | I think that **Soundproofing and quietness** for Gerontechnology and Spatial Integration, is |  |
| *I*_31_ | I think that **Color contrast for Gerontechnology** and Spatial Integration, is |  |
| *I*_32_ | I think that **Equitable use** for Gerontechnology and Spatial Integration, is |  |
| *I*_33_ | I think that **Flexibility in use** for Gerontechnology and Spatial Integration, is |  |
| *I*_34_ | I think that **Simple and intuitive use** for Gerontechnology and Spatial Integration, is |  |
| *I*_35_ | I think that **Perceptible feedback** for Gerontechnology and Spatial Integration, is |  |
| *I*_36_ | I think that **Tolerant of error** for Gerontechnology and Spatial Integration, is |  |
| *I*_37_ | I think that **Low physical effort** for Gerontechnology and Spatial Integration, is |  |
| *I*_38_ | I think that **Ease of access and use** for Gerontechnology and Spatial Integration, is |  |
| *I*_39_ | I think that **Mobility monitoring** for Gerontechnology and Spatial Integration, is |  |
| *I*_40_ | I think that **Emergency alerts** for Gerontechnology and Spatial Integration, is |  |
| *I*_41_ | I think that **Fire detection** for Gerontechnology and Spatial Integration, is |  |
| *I*_42_ | I think that **Wandering detection and prevention** for Gerontechnology and Spatial Integration, is |  |
| *I*_43_ | I think that **Smart information processing** for Gerontechnology and Spatial Integration, is |  |
| *I*_44_ | I think that **Heart rate monitoring** for Gerontechnology and Spatial Integration, is |  |
| *I*_45_ | I think that **Fall detection** for Gerontechnology and Spatial Integration, is |  |
| *I*_46_ | I think that **Activity detection** for Gerontechnology and Spatial Integration, is |  |
| *I*_47_ | I think that **Emergency button and touchscreen detection** for Gerontechnology and Spatial Integration, is |  |
| *I*_48_ | I think that **Automatic ambulance calling** for Gerontechnology and Spatial Integration, is |  |
| *I*_49_ | I think that **Automatic distress messaging** for Gerontechnology and Spatial Integration, is |  |
| *I*_50_ | I think that **Telehealth for preliminary diagnosis** for Gerontechnology and Spatial Integration, is |  |
| *I*_51_ | I think that **Recording of appointments and medication schedules** for Gerontechnology and Spatial Integration, is |  |
| *I*_52_ | I think that **Medical consultation via video call** for Gerontechnology and Spatial Integration, is |  |
| *I*_53_ | I think that **Social interaction via video call** for Gerontechnology and Spatial Integration, is |  |
| *I*_54_ | I think that **Daily and medication schedule tracking** for Gerontechnology and Spatial Integration, is |  |
| *I*_55_ | I think that **Touchscreen control panel** for Gerontechnology and Spatial Integration, is |  |

**Expert Profile** Name: Affiliation: Department/Position:

**Industry Category:** Industry / Government / Academia / Research Institution

**Gender:** Male / Female

**Education Level:** Associate degree / Bachelor’s degree / Master’s degree / Doctoral degree (Ph.D.)

**Age:** 25-30 years / 31–40 years / 41–50 years / 51–60 years / >60 years

**Years of Relevant Experience:** ≤3 years / 3–5 years / 5–10 years / >10 years

**Appendix 2. DEMATEL Questionnaire**

**Investigating the Relationship between**

**Gerontechnology and Spatial Integration Factors**

Dear Participant,

This survey investigates the relationship between gerontechnology and spatial integration factors. The objective of this project is to employ a questionnaire survey to examine the key factors of gerontechnology and spatial integration and to assess their interrelationships. The findings are expected to help prevent resource waste due to unplanned management and to provide critical decision-making support for relevant agencies in planning and design. This, in turn, will facilitate more efficient and evidence-based resource allocation for the development and subsequent management of intelligent spaces in suburban areas. Your valuable feedback will make a significant contribution to the outcomes and reliability of this research. The information you provide will be used exclusively for academic analysis and will remain strictly confidential.

1. **Descriptions of Core Dimensions and Associated Criteria**

(As shown in Table 4)

1. **Assessment of Interrelationships**
2. Within the dimension of ***Public Space***, the interrelationships among the indicators are :

| **Note.**  **Grey cells should not be completed.** | | | **Evaluation scale [0–4]** | | **0 = no influence; 1 = slight influence; 2 = moderate influence; 3 = strong influence; 4 = very strong influence.** | | |
| --- | --- | --- | --- | --- | --- | --- | --- |
| **Influence:**  **vertical axis on the horizontal axis** | Pavement maintenance and dedication | Pavement material, width and level | | Separation of bicycles from pavement | | Outdoor safety enhancement measures | Barrier-free access to and within buildings |
| Pavement maintenance and dedication |  |  | |  | |  |  |
| Pavement material, width and level |  |  | |  | |  |  |
| Separation of bicycles from pavement |  |  | |  | |  |  |
| Outdoor safety enhancement measures |  |  | |  | |  |  |
| Barrier-free access to and within buildings |  |  | |  | |  |  |

1. Within the dimension of ***Living Space***, the interrelationships among the indicators are :

| **Note.**  **Grey cells should not be completed.** | | **Evaluation scale [0–4]** | **0 = no influence; 1 = slight influence; 2 = moderate influence; 3 = strong influence; 4 = very strong influence.** | |
| --- | --- | --- | --- | --- |
| **Influence:**  **vertical axis on the horizontal axis** | Orientation and lighting | Roll-in shower adaptability | Indoor natural light access | Comfortable ambient temperature |
| Orientation and lighting |  |  |  |  |
| Roll-in shower adaptability |  |  |  |  |
| Indoor natural light access |  |  |  |  |
| Comfortable ambient temperature |  |  |  |  |

1. Within the dimension of ***Gerontechnology Application***, the interrelationships among the indicators are :

| **Note.**  **Grey cells should not be completed.** | | **Evaluation scale [0–4]** | **0 = no influence; 1 = slight influence; 2 = moderate influence; 3 = strong influence; 4 = very strong influence.** | | |
| --- | --- | --- | --- | --- | --- |
| **Influence:**  **vertical axis on the horizontal axis** | Emergency notification dispatch | Fire detection | Fall detection | Automatic transmission of distress messages | Appointment and medication schedule reminders |
| Emergency notification dispatch |  |  |  |  |  |
| Fire detection |  |  |  |  |  |
| Fall detection |  |  |  |  |  |
| Automatic transmission of distress messages |  |  |  |  |  |
| Appointment and medication schedule reminders |  |  |  |  |  |

1. The interrelationships among the evaluation ***Dimensions*** are :

| **Note.**  **Grey cells should not be completed.** | | **Evaluation scale [0–4]** | **0 = no influence; 1 = slight influence; 2 = moderate influence; 3 = strong influence; 4 = very strong influence.** | | |
| --- | --- | --- | --- | --- | --- |
| **Influence:**  **vertical axis on the horizontal axis** | Public Space | Living Space | | Age-Friendly Design | Gerontechnology Application |
| Public Space |  |  | |  |  |
| Living Space |  |  | |  |  |
| Age-Friendly Design |  |  | |  |  |
| Gerontechnology Application |  |  | |  |  |

**Expert Profile** Name: Affiliation: Department/Position:

**Industry Category:** Industry / Government / Academia / Research Institution

**Gender:** Male / Female

**Education Level:** Associate degree / Bachelor’s degree / Master’s degree / Doctoral degree (Ph.D.)

**Age:** 25-30 years / 31–40 years / 41–50 years / 51–60 years / >60 years

**Years of Relevant Experience:** ≤3 years / 3–5 years / 5–10 years / >10 years
